# Monosaccharide-Based Synthetic TLR4 Agonist Enhances Vaccine Efficacy against *Pseudomonas aeruginosa* Challenge

**DOI:** 10.1021/acsinfecdis.4c00932

**Published:** 2025-03-25

**Authors:** Maite Sainz-Mejías, Chaoying Ma, Yueran Hou, Irene Jurado-Martin, Alessio Romerio, Ana Rita Franco, Mohammed Monsoor Shaik, Julen Tomás-Cortázar, Francesco Peri, Siobhán McClean

**Affiliations:** † School of Biomolecular and Biomedical Sciences and Conway Institute of Biomolecular and Biomedical Research, University College Dublin, Belfield, Dublin 4 D04 V1W8, Ireland; ‡ Department of Biotechnology and Biosciences, University of Milano-Bicocca, Piazza della Scienza, 2, Milano 20126, Italy

**Keywords:** vaccine adjuvants, TLR4 agonist, infectious diseases, vaccines, *P. aeruginosa*, acute pneumonia, monosaccharide-based adjuvant

## Abstract

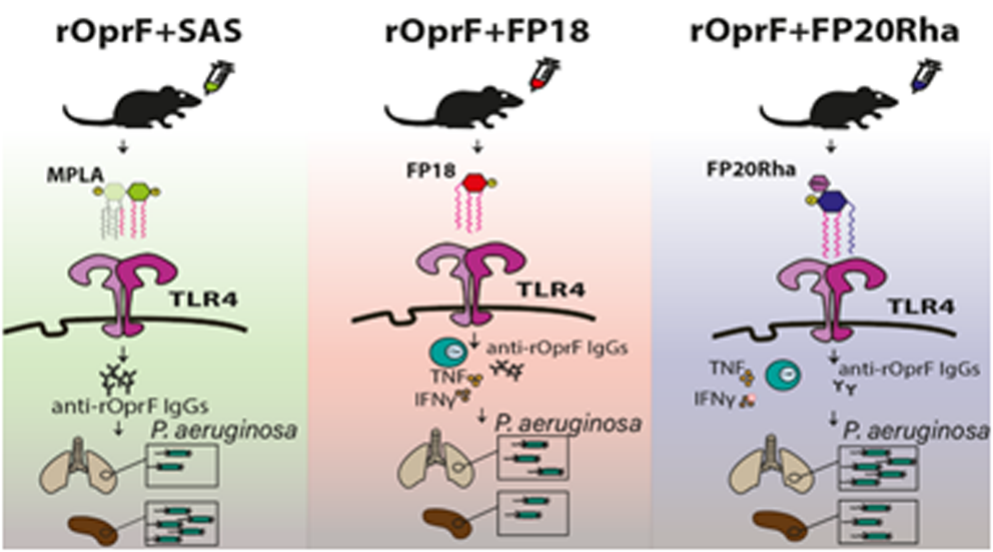

Vaccine adjuvants are critical to improve the immunogenicity, efficacy, and durability of vaccines; however, their development has lagged behind that of vaccine antigens. Monophosphoryl lipid A (MPLA), a clinically approved adjuvant that stimulates Toll-like receptor 4 (TLR4), faces manufacturing challenges due to its complex and long synthesis. With the aim of simplifying the structure of MPLA while retaining its biological activity, we developed monosaccharide-based molecules FP18 and FP20Rha that activate TLR4 signaling. Both TLR4 agonists induced robust antibody activity against the model antigen, ovalbumin. Here, we report the potential of these TLR4 agonists to enhance the protective efficacy of the well-characterized OprF antigen against *P. aeruginosa* infection. OprF adjuvanted with FP18 showed reduced bacterial loads in lungs and spleens, relative to antigen alone in an acute *P. aeruginosa* pneumonia model. FP18-adjuvanted OprF also enhanced the production of anti-OprF antibodies and stimulated IFNγ and TNF in CD4^+^ T cells, suggesting a Th1-skewed cellular immune response. These adjuvants have promise for accelerating the development of effective vaccines against *P. aeruginosa* and other infectious diseases.

Adjuvants play a vital role in vaccine development. They are critical in tailoring subunit vaccine responses by enhancing immune responses and the duration of the response through a variety of mechanisms. Adjuvants stimulate innate immunity by directly interacting with pattern recognition receptors or inducing cellular stress, leading to the release of immunostimulatory and inflammatory signals. They facilitate the maturation of antigen-presenting cells (APCs), which improves the interaction between the APCs and T cells. This leads to a more robust and diverse production of T helper-polarizing cytokines, multifunctional T cells, and increased levels of antibodies. As a result, adjuvanted vaccines offer stronger, more durable immunity while reducing the necessary antigen dose and the frequency of boosters, making them more efficient and cost-effective^1,2^ However, despite their pivotal role, vaccine adjuvant development has dramatically lagged behind that of vaccine antigen development. Alum, discovered in 1926, was the only vaccine adjuvant licensed for use in humans for over 70 years, until the approval of MF59 in 1997.^2−4^ Although there has been some development of vaccine adjuvants in the past 20 years, the number of effective adjuvants available with low human toxicity is still limited.^1,3,5^ Monophosphoryl lipid A (MPLA), a detoxified form of lipopolysaccharide (LPS), activates Toll-like receptor (TLR4) on antigen-presenting cells (APCs), triggering nuclear factor kappa-light-chain-enhancer of activated B cells (NF-κB) activation and pro-inflammatory cytokine expression, thereby driving T-helper (Th)1 cell responses.^6^ MPLA is a component of the alum-containing AS04 adjuvant system used in licensed human papillomavirus^7^ and hepatitis B vaccines.^6,8^ Combined with QS-21 in systems such as AS01, AS02, and AS15, MPLA has also demonstrated efficacy against diverse infectious diseases and cancers.^6,9^ MPLA’s clinical success in licensed vaccines highlights the potential of TLR agonists as effective adjuvants in vaccination strategies.^10−14^ However, MPLA manufacturing is complex. Synthetic MPLA (named GLA, produced by Avanti Lipids) requires a long and challenging synthesis, while MPLA obtained by chemical transformation (dephosphorylation) of natural lipid A lacks chemical homogeneity.^11,15^ Consistent quality, stringent regulatory standards, and scaling up of production for global demand present logistical and economic hurdles, which impact vaccine accessibility and affordability.^2,3^ Furthermore, the variability in different forms of MPLA and the difficulty in obtaining clinical-grade MPLA contribute to inconsistencies in vaccine studies across laboratories.^16^

To address MPLA’s limitations, a series of TLR4 ligands composed of a glucosamine core functionalized with a phosphate ester and three fatty acid chains of variable length (10–14 carbons) were developed (Figure 1).^11,17^ One of these molecules, FP18, binds to the TLR4/MD-2 dimer with submicromolar affinities, stabilizing its active form and activating both MyD88- and TRIF-dependent TLR4 signaling pathways along with the NOD-like receptor family pyrin domain-containing 3 (NLRP3) inflammasome. Its 7-step synthesis is significantly shorter than that of MPLA (over 25 steps), resulting in higher industrial scalability and reduced production costs.^11^ A second generation of TLR4 ligands, the FP20 series, are triacylated glucosamine derivatives where the phosphate group at C1 is relocated to C4, thus enhancing their chemical stability (Figure 1).^17^*In vitro*, FP20 did not induce NF-κB or phosphorylated interferon regulatory factor 3 (p-IRF-3) nuclear translocation but activated mitogen-activated protein kinase (MAPK) pathways and promoted NLRP3-dependent inflammasome activation.^17^ FP20Rha, a variant incorporating an l-rhamnose unit at the C6 carbon to mimic the first sugar of the LPS core, showed enhanced antibody production against the model antigen, ovalbumin.^18,19^ Overall, neither FP18 nor FP20Rha showed any toxicity in mouse immunization experiments with ovalbumin, and both induced robust antigen-specific IgG production, thus indicating promising adjuvant activity.^17,18^ However, whether these molecules can support protective immune responses that lead to effective prophylactic vaccines, compared to more complex counterparts like formulations containing MPLA, has yet to be established.

**Figure 1:**
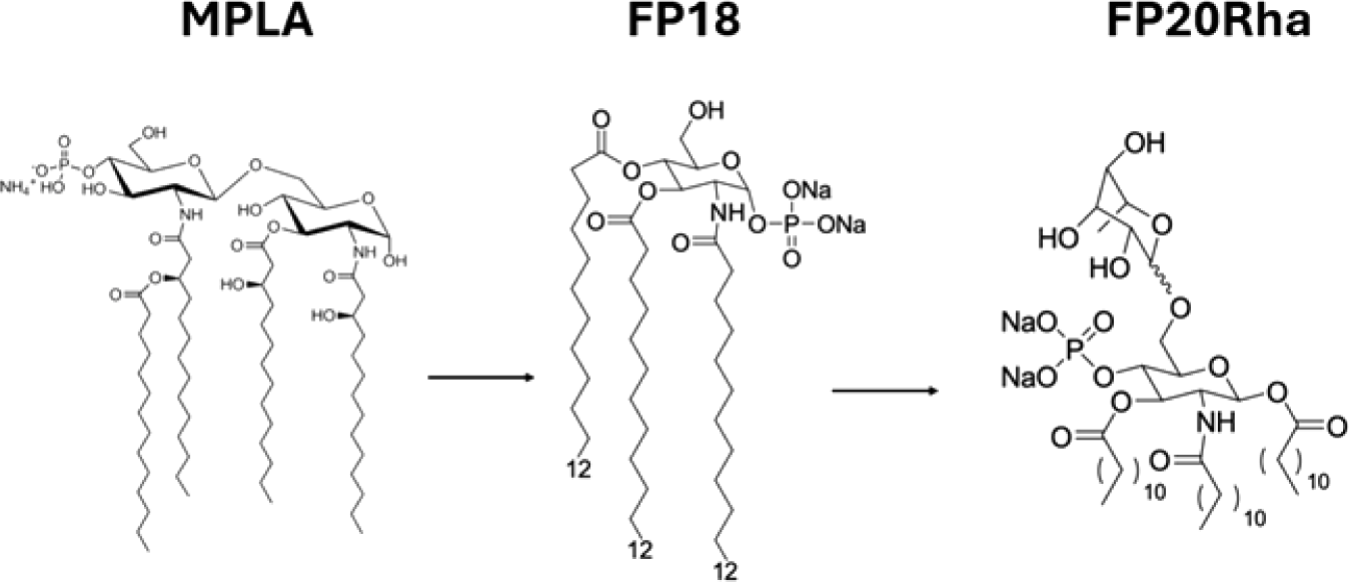
Chemical structure of monophosphoryl lipid A (MPLA) and the FP compounds used in the study: FP18 and FP20Rha.

The recent WHO priority list highlights carbapenem-resistant *P. aeruginosa* as a critical global health threat.^20^ Despite extensive efforts, no licensed vaccine exists against this pathogen. OprF, a leading antigen candidate for *P. aeruginosa* vaccines, has been extensively evaluated alone or fused with OprI in a variety of vaccine formats. It was protective in multiple preclinical studies but failed in a non-adjuvanted form in human phase II/III trials.^21−26^ OprF is distinguished as a robust antigen for evaluating novel adjuvants, as it has been well-characterized. To evaluate the protective potential of FP18 and FP20Rha compounds in prophylactic vaccines, we investigated the protective effect of immunization with rOprF adjuvanted with either FP18 or FP20Rha in a murine model of acute *P. aeruginosa* pneumonia, comparing them with unadjuvanted rOprF or with rOprF combined with another MPLA-containing adjuvant, the Sigma Adjuvant System (SAS).

## Results

### Immunization with OprF Adjuvanted with FP Molecules Induced a Better Reduction in Bacterial Bioburden and/or Dissemination to the Spleen than OprF Alone

**Figure 2:**
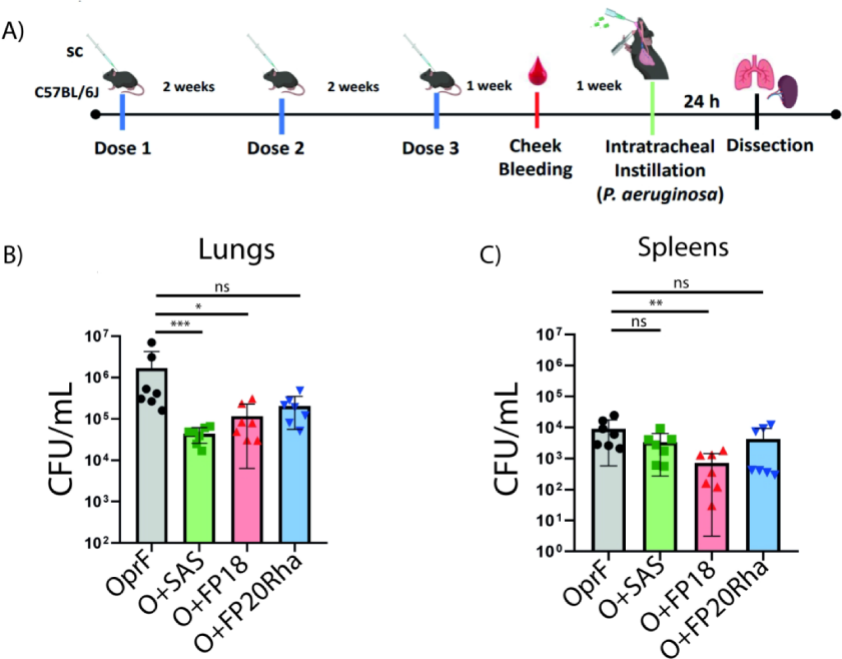
Bacterial lung colonization and dissemination to spleens. (A) Timeline of immunization (50 μg rOprF alone; 50 μg rOprF plus 25 μg FP18; 50 μg rOprF plus 25 μg FP20Rha or 50 μg rOprF plus SAS (∼25 μg of active component) in 100 μL), blood collection, and *P. aeruginosa* challenge. (B,C) Bacterial clearance after immunization with rOprF adjuvanted with FP18 and FP20Rha or SAS adjuvant. Immunization of OprF in combination with SAS and FP18 significantly decreased *P. aeruginosa* colonization in the lungs by 1.5 log and 1.15 log, respectively (Kruskal–Wallis test, *p* = 0.003 and *p* = 0.026). rOprF + FP18 significantly decreased bacteria dissemination to the spleens relative to the immunization with antigen alone (Kruskal–Wallis test, *p* = 0.003). Each point represents a single mouse (*n* = 7) and the mean ± SD.

To examine whether FP18 and FP20Rha molecules could enhance the protective efficacy of rOprF against a*P. aeruginosa* challenge, they were assessed in a *P. aeruginosa* acute pneumonia model in C57BL/6J mice (Figure 2A). The rOprF identity was confirmed by LC-MS, and endotoxin content was determined (0.20 EU/dose) in line with previous experiments^11^ (Table S1). Bacterial lung burden was significantly decreased by 1.15 log_10_ CFU (*p* = 0.026) in the group immunized with rOprF + FP18 relative to rOprF alone (Figure 2B), indicating that FP18 can enhance the protective immune response against *P. aeruginosa*. Moreover, the reduction in bacterial bioburden was comparable to the group immunized with rOprF + SAS (1.5 log_10_ CFU reduction, *p* = 0.003), indicating that FP18 was almost as effective as SAS when used as an adjuvant (Figure 2B). Immunization with rOprF + FP20Rha showed a 0.91 log_10_ CFU reduction in bacterial load, but the bioburden reduction was not statistically significantly changed relative to the group that received antigen alone (*p* = 0.417). Immunization with rOprF + FP18 significantly reduced bacterial dissemination to the spleen by 1.09 log_10_ CFU (*p* = 0.003) relative to mice immunized with OprF alone, which was superior to OprF adjuvanted with either SAS or FP20Rha, as these formulations did not show any significant reduction in spleen dissemination relative to rOprF alone (0.43 and 0.32 log_10_ CFU, respectively, Figure 2C).

Notably, neither FP18 nor FP20Rha showed any apparent detrimental effect on the mice, as evidenced by a lack of weight loss (Figure S4). In contrast, immunization with rOprF + SAS caused more weight loss over the first 5 days postimmunization or postboosts than immunization with rOprF alone or FP18- and FP20Rha-adjuvanted groups (Figure S4). All the mice recovered and gained weight after the completion of all immunizations. There were no significant differences in weight loss after the bacterial challenge between the groups (Figure S4).

### Immunization with FP18-Adjuvanted rOprF Stimulated Stronger Serological Responses than FP20Rha and OprF Alone

**Figure 3:**
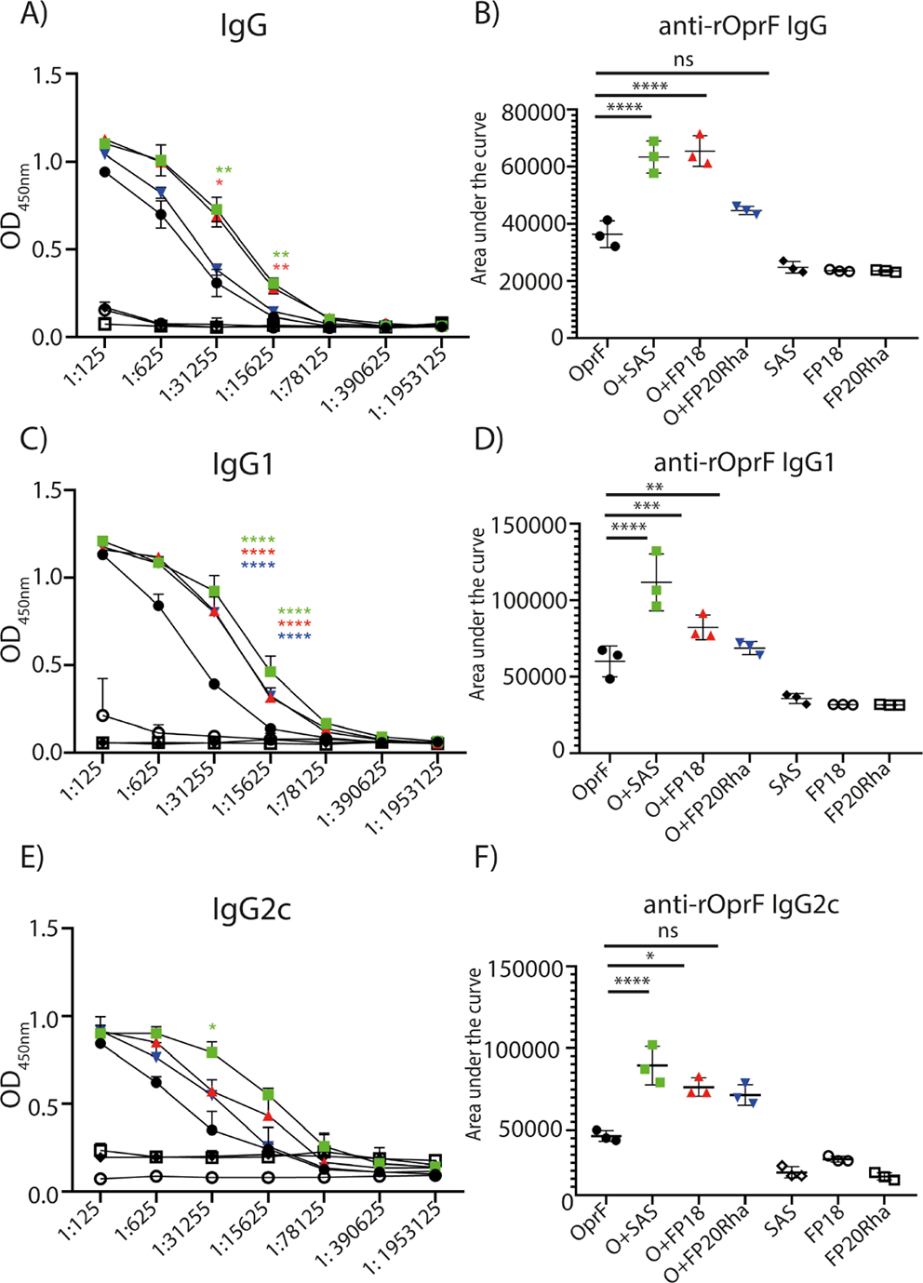
Seroconversion in response to adjuvanted OprF at 35 days post-immunization. Determined by ELISA of rOprF-specific IgGs in sera collected from immunized groups: rOprF (black circles), OprF + SAS (green squares), rOprF + FP18 (red triangles) and rOprF + FP20Rha (blue triangles). Values represent mean OD ± SD (A,C,E) or AUC ± SD (B,D,F). The sera of the seven mice were pooled and technical triplicates were performed (A,C,E). The AUC of each technical triplicate was calculated and is represented as a single point in the graphs (B,D F).

To determine whether the adjuvants enhanced specific humoral responses against rOprF, we collected serum 1 week after the second booster, and antigen-specific total IgG, IgG1, and IgG2c levels were determined. Groups immunized with rOprF + SAS or rOprF + FP18 (*p* < 0.0001) expressed higher levels of antigen-specific total IgG relative to the group immunized with rOprF + FP20Rha or rOprF antigen alone (Figure 3A,B). In addition, antigen-specific IgG1 levels were higher in all the groups immunized with adjuvanted rOprF relative to the group immunized with rOprF alone but highest in the SAS- and FP18-adjuvanted groups (Figure 3C,D). These latter two groups also showed significantly more antigen-specific IgG2c levels than rOprF alone (Figure 3E,F). The calculated IgG1/IgG2c ratio (Table S3) highlighted differences in the immune response across groups. For rOprF and rOprF + SAS, the IgG1/IgG2c ratio was 1.0, suggesting a balanced Th1/Th2 response, whereas for OprF + FP18 and OprF + FP20Rha, the ratio was 5, suggesting a strong Th2 response.

### Immunization of rOprF Adjuvanted with FP Compounds Increased the Levels of CD4^+^ but Not CD8^+^ T Cells

**Figure 4:**
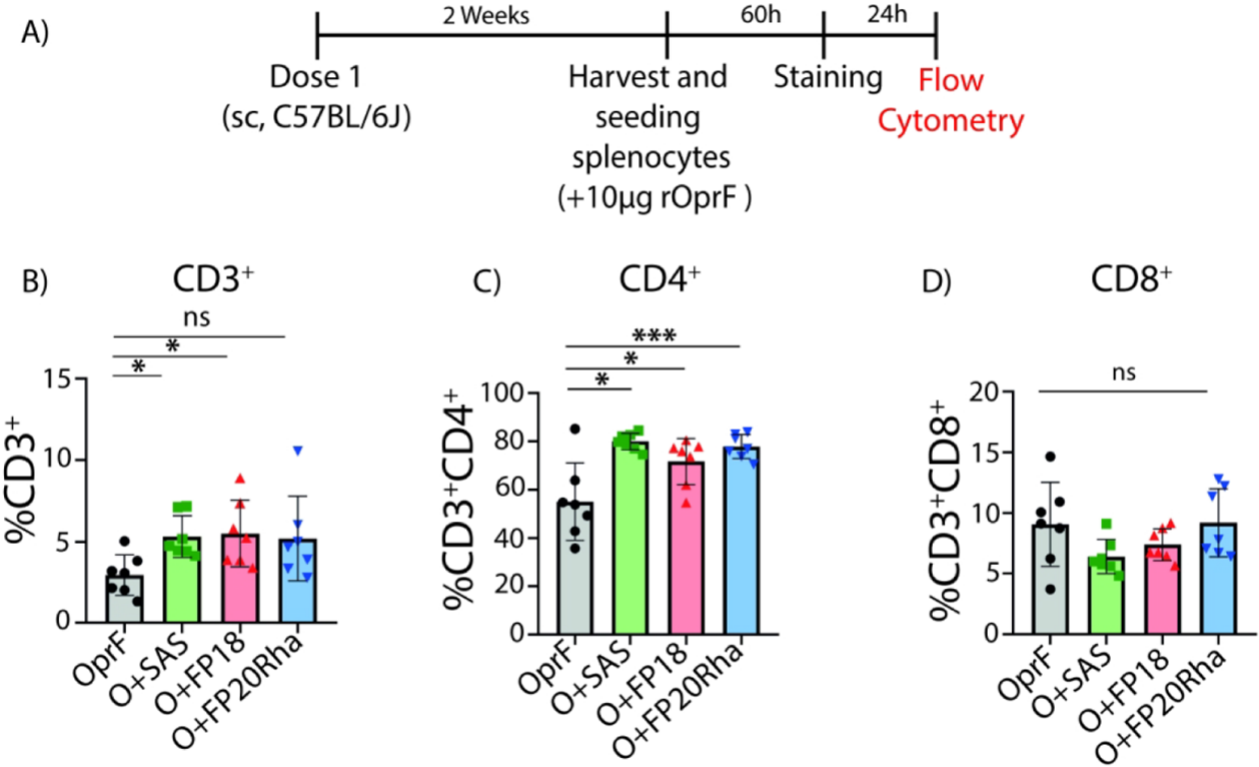
(A) Schematic illustration of the experimental timeline of the mouse study. Spleens were aseptically collected, disrupted, and filtered to obtain single-cell suspensions. One million splenocytes per well (in triplicate) were cultured with 10 μg/mL OprF for 60 h in RPMI supplemented with 10% FBS and 1% penicillin–streptomycin. (B) Percentages of singlet cells expressing the CD3 marker. (C) Percentages of parent cells expressing the CD4 marker. (D) Percentages of parent cells expressing the CD8 marker. (B,C,D) Each point in the bar charts represents the mean of technical triplicates of splenocytes from individual mice (*n* = 7) as follows: splenocytes from mice immunized with rOprF (black circles), rOprF + SAS (green squares), rOprF + FP18 (red triangles), or rOprF + FP20Rha (blue triangles). Asterisks denote statistically significant differences: *p < 0.05; **p < 0.01; ***p < 0.001.

Splenocytes collected from mice that received a single subcutaneous immunization with rOprF alone or SAS-, FP18-, or FP20Rha-adjuvanted rOprF were restimulated with 10 μg/mL rOprF to assess antigen-specific T-cell recall responses by flow cytometry (Figure 4A). Immunization with OprF adjuvanted with SAS or FP18 led to significantly higher levels of CD3^+^ T cells (*p* < 0.05) relative to immunization with rOprF alone or to immunization with rOprF adjuvanted with FP20Rha (Figure 4B). Mice immunized with rOprF and any of the three adjuvants showed significantly more CD4^+^ T cells relative to immunization with antigen alone (Figure 4C). However, no increase in CD8^+^ T-cell numbers was observed in any of the groups (Figure 4D).

### Immunization of rOprF Adjuvanted with FP Compounds Increased the Levels of Activation in CD4^+^ T Cells Relative to Immunization with Antigen Alone

**Figure 5:**
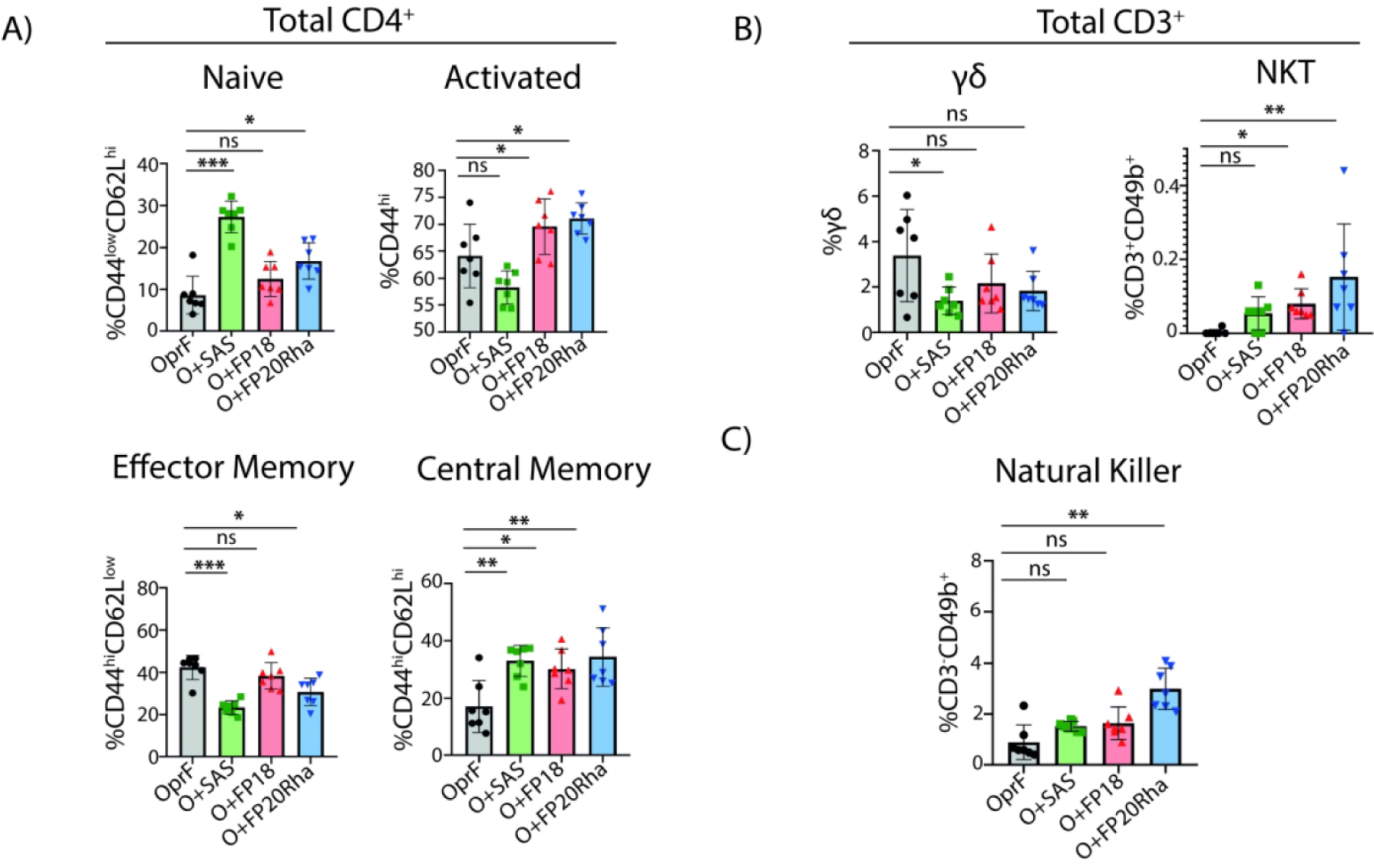
Subpopulations of T cells from restimulated splenocytes after immunization with rOprF adjuvanted with FP compounds. (A) Percentages of different populations of CD3^+^CD4^+^ T cells defined by different levels of CD44 and CD62L markers: CD44^low^ CD62L^hi^, naïve; total CD44^hi^, activated; CD44^hi^CD62L^low^, effector memory, and CD44^hi^ CD62L^hi^; central memory. (B) Percentages of gamma-delta T cells and NKT cells. (C) Percentages of natural killer (NK) cells based on their expression of CD49b marker. Asterisks denote statistically significant differences: *p < 0.05; **p < 0.01; ***p < 0.001. Each point in the graphs indicates the mean of technical triplicates of splenocytes from individual mice (*n* = 7): splenocytes from mice immunized with rOprF (black circles); rOprF + SAS (green squares); rOprF + FP18 (red triangles); or rOprF + FP20Rha (blue triangles).

Both FP adjuvants were previously characterized in studies incorporating OVA as an adjuvant to examine the mechanism of action and to highlight their selectivity as TLR4 ligands.^11,17,19^ To examine the cellular responses in the context of OprF as the immunizing antigen, we examined the recall responses elicited in all immunized mouse groups. When subpopulations of CD4^+^ T cells were classified by flow cytometry, it was apparent that immunization with either rOprF + SAS or rOprF + FP20Rha showed higher levels of naïve T cells relative to immunization with antigen alone, while rOprF + FP18 showed comparable levels to rOprF-only immunized mice (Figure 5A). Mice immunized with OprF adjuvanted with FP18 or FP20Rha showed higher levels of activated CD4^+^ T cells (CD4^+^ CD44^hi^) relative to immunization with either antigen alone or OprF + SAS (*p* < 0.01). No group showed higher levels of effector memory T cells (CD4^+^ CD44^hi^CD62L^low^ T_EM_), relative to immunization with antigen alone (Figure 5A). In contrast, all mice immunized with rOprF and any of the adjuvants showed higher levels of central memory T cells (CD4^+^ CD44^hi^CD62L^hi^ T_CM_, *p* = 0.0033, OprF + SAS, OprF + FP20Rha, and *p* = 0.0015) (Figure 5A).^27,28^

**Figure 6:**
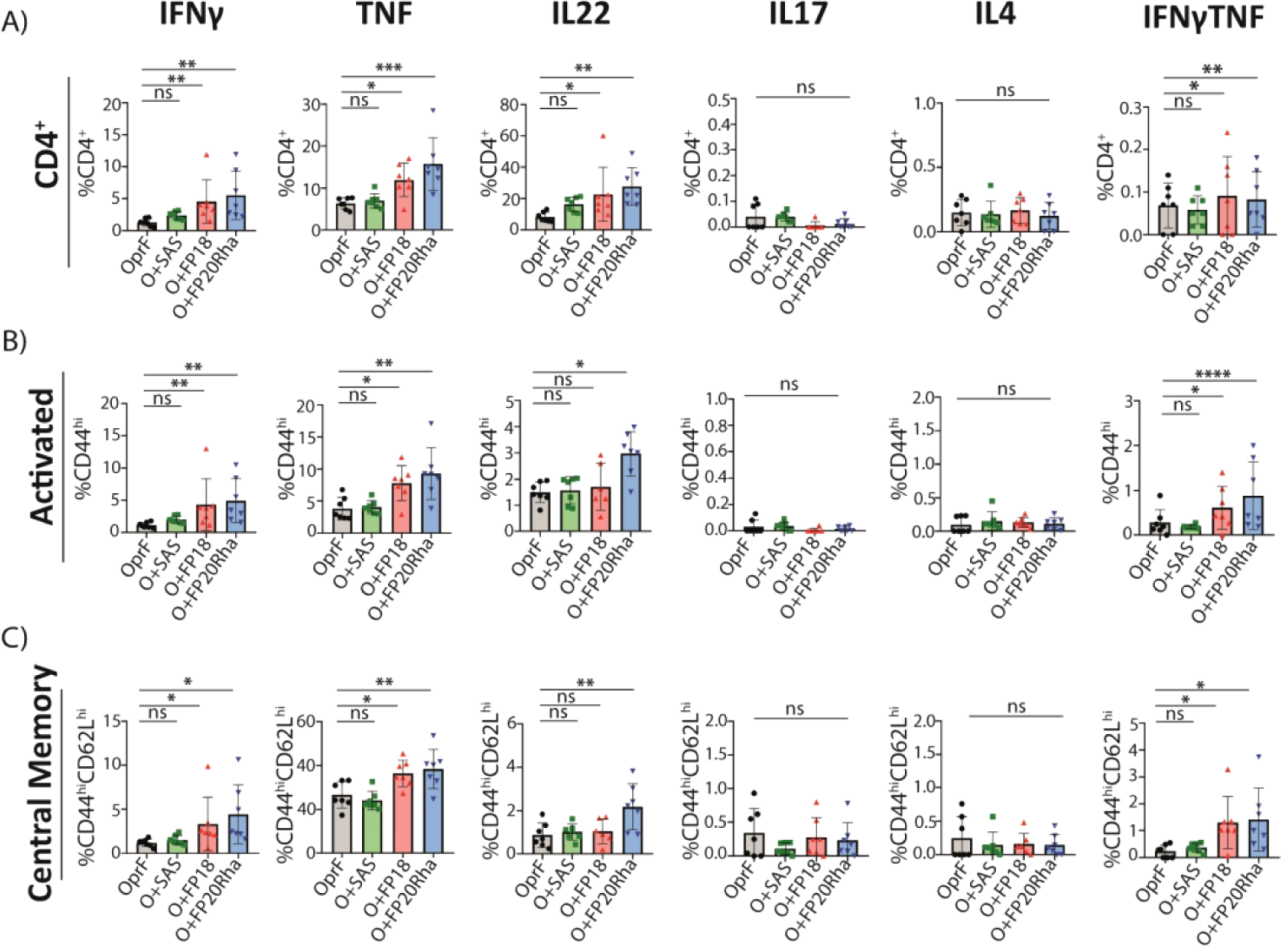
Evaluation of the cytokines elicited by CD4^+^ T-cell subtypes in restimulated splenocytes. (A) Total CD4^+^ T cells expressing IFNγ, TNF, IL22, IL17, or IL4 cytokines. (B) Percentages of activated CD4^+^ T cells expressing IFN-γ, TNF, IL22, IL17, or IL4. (C) Percentages of central memory CD4^+^ T cells expressing IFNγ, TNF, IL22, IL17, or IL4. Asterisks denote statistically significant differences: *p < 0.05; **p < 0.01; ***p < 0.001. Each point in the graphs indicates the mean of technical triplicates of splenocytes from individual mice (*n* = 7) as follows: splenocytes from mice immunized with rOprF (black circles); rOprF OprF + SAS (green squares); rOprF + FP18 (red triangles); or rOprF + FP20Rha (blue triangles).

Gamma-delta (γδ) T cells play an important role in regulating the initial immune response to lung infections caused by various bacterial pathogens, such as *Mycobacterium tuberculosis*, *Streptococcus pneumoniae*, or *Staphylococcus aureus*.^29,30,31^ Hence, we assessed whether combining rOprF with FP18 or FP20Rha stimulated the γδ T-cell population, but no effect was observed (Figure 6B). Furthermore, natural killer T (NKT) cells play a critical role in microbial infections, and NKT cells have been shown to contribute to *P. aeruginosa* clearance from the lungs of mice.^32,33^ Natural killer (NK) cells are also involved in the defense against respiratory bacterial pathogens, and it was recently reported that NK cells kill extracellular *P. aeruginosa*.^34^ Consequently, we evaluated both populations and observed that NKT (CD3^+^CD49b^+^) cells were elevated after immunization with rOprF adjuvanted with FP18 (*p* = 0.0238) or FP20Rha (*p* = 0.0011) (Figure 5B), while levels of natural killer (NK) cells (CD3^–^CD49b^+^) were higher after immunization with OprF adjuvanted with FP20Rha (*p* < 0.0001, Figure 5C), relative to immunization with antigen alone.

### Immunization of rOprF Adjuvanted with FP Compounds Increased the Levels of IFNγ and IL22-Producing CD4^+^ T Cells but Not IL4 or IL17 in C57BL/6J Mice

Cellular cytokine responses contribute to protection against *P. aeruginosa*,^4^ therefore to determine the cellular immune profiles elicited by the TLR4 agonists, we evaluated the expression of IFNγ and TNF as indicators for Th1 responses, IL4 for Th2 responses, IL17 for Th17 responses, and IL22 for Th22 responses (Figure 6). Immunization with rOprF + FP18 or rOprF + FP20Rha showed significantly higher levels of IFNγ production by CD4^+^ and activated CD4^+^(CD44^hi^) cells relative to immunization with rOprF alone (*p* < 0.01). Mice immunized with rOprF adjuvanted with FP18 or FP20Rha showed higher levels of IFNγ elicited by T_CM_ cells (*p* < 0.05) among the splenocyte populations. SAS-adjuvanted rOprF showed comparable levels of IFNγ and TNF in all the populations examined relative to the rOprF alone control group. Splenocytes from mice immunized with either rOprF with FP18 or rOprF with FP20Rha showed significantly higher levels of TNF in CD4^+^, CD4^+^ CD44^hi^, and T_CM_ cells relative to immunization with rOprF alone (*p* < 0.01), while immunization with rOprF adjuvanted with SAS did not enhance TNF production in any of the CD4^+^ T-cell subpopulations examined, relative to immunization with antigen alone (Figure 6). The groups immunized with rOprF adjuvanted with FP18 or FP20Rha significantly augmented IL22 production relative to the control CD4^+^ T cells. In addition, rOprF + FP20Rha showed higher levels of IL22 in CD4^+^ CD44^hi^ and T_CM_ cells relative to the antigen alone. Recombinant OprF adjuvanted with SAS showed no significant production of IL22 relative to rOprF alone. Furthermore, mice immunized with rOprF adjuvanted with FP18 and FP20Rha showed a higher dual production of IFNγ-TNF cytokines from CD4^+^, CD4^+^CD44^hi^, and CD4^+^CD62L^hi^CD44^hi^ T cells relative to immunization with antigen alone. No group showed any production of IL4 or IL17, characteristic of Th2 or Th17 responses, respectively. Minimal cytokines were produced by effector memory T cells (Figure S17). Overall, the cytokine profile suggested a Th1-biased response for the FP18 adjuvant and a Th1/Th22-skewed response for FP20Rha using rOprF as the model antigen (Figure 6).

**Figure 7:**
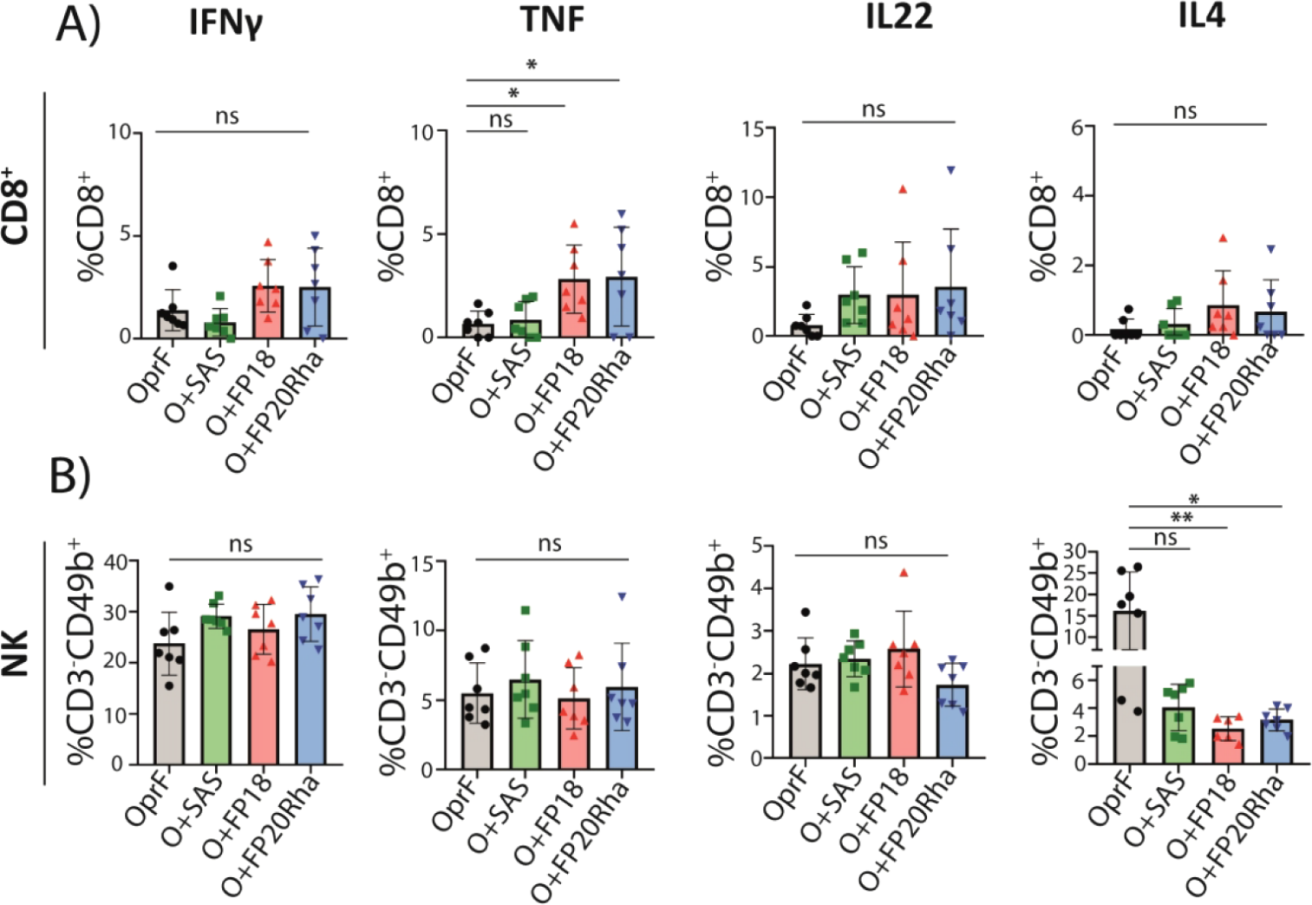
Evaluation of the cytokines elicited by CD8^+^ and NK cells in spleens after immunization with FP compounds. (A) Total CD8^+^ T cells expressing IFN-γ, TNF, IL22, or Il4 cytokines. (B) Percentages of NK cells expressing IFN-γ, TNF, IL22 or IL4 cytokines. Asterisks denote statistically significant differences: *p < 0.05; **p < 0.01; ***p < 0.001. Each point in the graphs indicates the mean of technical triplicates of splenocytes from individual mice (*n* = 7) as follows: splenocytes from mice immunized with rOprF (black circles); rOprF + SAS (green squares); rOprF + FP18 (red triangles); or rOprF + FP20Rha (blue triangles).

All immunized mice showed comparable IFNγ, IL22, IL-17, and IL4 production in CD8^+^ T cells (Figure 7A). Immunization with rOprF adjuvanted with FP18 or FP20Rha showed higher levels of TNF by CD8^+^ T cells (*p* < 0.05) relative to mice that received antigen alone (Figure 7A). The levels of IL4 in NK cells were lower in the groups adjuvanted with FP18 and FP20Rha compared with the control group. No significant differences were observed in any other evaluated cytokines (Figure 7B).

## Discussion

The development of effective vaccines against bacterial infections and the pressing threat of antimicrobial resistance demand the creation of safe and potent adjuvants.^3,35,36^ TLR4 agonists have emerged as promising candidates for enhancing the protective effect of vaccines against bacterial pathogens; however, existing TLR4 agonists such as MPLA pose significant challenges due to their complex synthesis.^11,14^ In this context, FP18 and FP20Rha represent a breakthrough, offering simpler synthesis strategies that enhance industrial scalability, reduce waste, and lower costs.^11,17^

Both glucosamine-derived adjuvants have previously shown promise as adjuvants for prophylactic vaccines by enhancing the production of OVA-specific antibodies in immunized mice.^17,18^ However, it is crucial that these adjuvants not only stimulate the immune system but also support prophylactic responses against infection *in vivo*. Here, we evaluated for the first time their ability to enhance the protective effect in an infection scenario using a well-characterized antigen, OprF. The reduction of bacterial colonization achieved with rOprF + FP18 in a *P. aeruginosa* acute pneumonia infection model was comparable to that observed with rOprF adjuvanted with SAS, a more complex adjuvant which, to date, lacks a human safety profile. Notably, rOprF + FP18 was the only adjuvant that resulted in reduced bacterial dissemination to the spleen, indicating its potential to lessen disease severity as well as colonization. Additionally, mice immunized with FP18 or FP20Rha did not exhibit adverse reactions or weight loss, suggesting a potentially safer profile than SAS, though further preclinical safety evaluations are necessary.

All three adjuvants increased IgG1 production, but only rOprF + SAS and rOprF + FP18 enhanced IgG2c production. A higher IgG1/IgG2 ratio is suggestive of Th2-biased responses, while a higher IgG2/IgG1 ratio suggests a Th1-skewed response, shown to be beneficial in previous *P. aeruginosa* vaccine formulations.^25,37^ The IgG profile in OprF-immunized mice aligns with previous analyses of FP compounds during OVA immunization.^17,18^ Notably, the FP compounds elicited a higher IgG1/IgG2 ratio, indicating a Th2-biased response, while the SAS adjuvant promoted a more balanced Th1/Th2 antibody response. We used the subcutaneous route for immunization, in line with previous findings where OVA was both safe and effective in eliciting humoral responses.^17^ This study provides the first *in vivo* profile of cellular responses to novel FP compounds. Immunization with rOprF combined with any of the evaluated adjuvants enhanced the levels of CD4^+^ T cells while maintaining comparable levels of CD8^+^ T cells. Subunit protein vaccine antigens have limited access to the cytosol, which is crucial for MHC class I presentation and CD8^+^ T cell activation. Consequently, the lack of CD8^+^ enhancement aligns with previous findings reported for subunit vaccines.^38,39^

Optimal vaccines generate a balanced population of multifunctional T cells, including effector and central memory T cells capable of rapid responses and sustained memory. OprF adjuvanted with any of the three evaluated adjuvants enhanced the levels of central memory T cells, but none of the adjuvanted groups enhanced effector memory T-cell numbers. This could be explained by the fact that T_EM_ cells circulate in blood and tissues, while T_CM_ cells primarily reside in lymphoid tissue.^40^ CD44, a key activation marker, is rapidly upregulated following antigen exposure and maintained in memory T cells.^41,42^ T_CM_ cells are characterized by the expression of the homing receptor CD62L (CD4^+^CD44^hi^CD62L^low^) whereas T_EM_ cells lack CD62L (CD4^+^CD44^hi^CD62L^low^).^40^ Previous studies have shown that TCR stimulation induces the rapid shedding of CD62L from the T-cell surface by proteolytic cleavage, but within 24–48 h, CD62L is re-expressed,^43,44^ thus the T_EM_ and T_CM_ characteristic markers may be observed in short-term studies. However, given the limited antigen exposure of the evaluated cells and the time of the study, further long-term studies are necessary to confirm whether the adjuvants effectively enhance T-cell memory, as memory involves a complex reprogramming of the cells beyond the evaluated time and the studied markers.^45^

Multifunctional T cells producing more than a single cytokine have been correlated with protective vaccinations.^46,47^ For instance, the co-production of IFNγ and TNF enhances the killing of pathogens like *Leishmania major* and *Mycobacterium tuberculosis* more effectively than either cytokine alone.^48,49^ Here, FP18 and FP20Rha both boost the production of IFNγ and TNF by CD4^+^, CD4^+^CD44^hi^, and CD4^+^CD62L^hi^CD44^hi^ T cells, relative to immunization with rOprF alone or adjuvanted with SAS. This may explain the superior reduction in dissemination to the spleen in the rOprF + FP18 group, compared to the relatively limited protection against bacterial dissemination provided by the SAS-adjuvanted group. Surprisingly, FP20Rha was not protective despite eliciting strong inflammatory responses,^46^ which may be due to FP20Rha's limited stimulation of total IgG and IgG2c antibodies relative to FP18 or SAS, resulting in lower bacterial clearance. This suggests that an optimal balance between humoral and cellular responses is crucial for effective protection against an acute infection. An important difference between FP18 and FP20, the parent molecule of FP20Rha, is the inability of FP20 to induce the translocation of the p-NF-κB p65 subunit or p-IRF-3, so this mechanism of action was attributed to the MAPK pathway as it induced p38 MAPK.^17^ This may explain, at least in part, the strong stimulation of TNF after immunization with OprF adjuvanted with FP20Rha, without robust humoral responses. The stimulation of NF-κB might be higher after immunization with FP18, as suggested in previous publications,^11^ giving a better balance of cellular and humoral responses. NF-κB pathways are required for complete mitogen-induced B-cell proliferation and survival.^50^ The stimulation of IFNγ and TNF and the lack of IL4 suggest a potent skewed Th1 response by both FP18 and FP20Rha, contrary to what was suggested by the IgG1/IgG2c ratio. This was also observed in a previous evaluation of the IC43 vaccine against *P. aeruginosa* with high IgG1 production, at the same time as a high IFNγ stimulation, mainly indicative of a Th2/Th1 response.^51^ Furthermore, FP20Rha enhances IL22 production but not IL17A. In a previously reported *P. aeruginosa* pneumonia model, IL22 production was negatively correlated with lung neutrophil recruitment, despite increased susceptibility to infection and lung damage with higher neutrophil accumulation being observed.^52^ In a vaccine context, this anti-inflammatory mechanism might be detrimental, as shown by the minimal reduction in bacterial colonization and dissemination in the FP20Rha-adjuvanted group. Despite inducing stronger humoral responses, the cytokine response elicited by SAS was generally muted compared to those of FP18 and FP20Rha. SAS, a squalene-based formulation, includes additional components, such as surfactants and mycobacterial cell wall components. These elements may have contributed to enhanced humoral responses without skewing the cellular response toward a specific cytokine profile.

A potential limitation of the study is the lack of survival data as indicators of protection. These were not conducted due to 3R considerations in the context of their severity. However, the observed reduction in bacterial colonization and dissemination with FP18-adjuvanted antigen suggests it is a promising candidate for enhancing survival against infection. This positions FP18 as a promising alternative to vaccines containing MPLA or for studying new antigens with well-defined, safe adjuvant formulations. FP18 is now available in the Avanti Lipids catalog. The COVID-19 pandemic underscores the urgent need for scalable and effective vaccine platforms, where adjuvants like FP18 will play a critical role in combating antimicrobial resistance and enhancing public health preparedness. Moreover, the use of FP18 in research paves the way for superior consistency, enhancing the reproducibility of results across laboratories.

## Conclusions

We have evaluated two novel TLR4 adjuvants and shown that FP18 has potential as an adjuvant in prophylactic vaccines. Immunization with FP18 demonstrated several key benefits: (i) bacterial clearance in the lung, (ii) reduced bacterial dissemination to the spleen, (iii) higher levels of IgG1 and IgG2c anti-rOprF antibodies, and (iv) increased production of proinflammatory cytokines. Developing novel, improved adjuvants is challenging, but our study shows that rational development, combined with previously identified antigens, can lead to effective vaccine formulations. This is the first study demonstrating the effectiveness of the novel adjuvant FP18 in an *in vivo* infection model. While focused on *P. aeruginosa*, FP18 may also be suitable for other vaccine formulations against ESKAPE pathogens. Incorporating easily scalable molecules into vaccine formulations will be crucial for enabling non-marketable vaccine candidates to reach licensing.

## Materials and Methods

### Ethical Statement

All the work involving the animals was approved by the UCD Animal Research Ethics Committee (AREC-21-19), and mice were maintained according to the regulations of the Health Products Regulatory Authority (Directive 2010/63/EU and Irish Statutory Instrument 543 of 2012), authorization number AE18982/P209.

### Recombinant OprF_his Expression

Genomic DNA was extracted from the *P. aeruginosa* PAO1 strain genome (GB: AE004091), and the *oprf* sequence was amplified by PCR with these specific primers: 5’-CGCGGATCCAAACTGAAGAACACCTTAGGCGTTGTC-3’ (Fw) and 5’-CCCAAGCTTTTACTTGG-CTTCGGCTTCTACTTCGGC-3’ (Rev). The *oprf* gene fragment was cloned into the pET28a expression vector (Novagen). The recombinant plasmid was then sequenced, and the sequence was compared with the sequence reported in GenBank (Gene ID: 878442) using SnapGene software 7.2 (https://www.snapgene.com). The 6xHis tag was located at the N-terminal domain (Figure S1). Plasmids were purified using a Plasmid Miniprep Kit (Qiagen) according to the manufacturer’s protocol. FastDigest restriction enzymes (BamHI and HindIII) and a Rapid DNA Ligation Kit (Thermo Scientific) were used according to the manufacturer’s instructions. *E. coli* BL21 (DE3) cells (Qiagen) were made chemically competent and transformed with the resulting plasmid pET28a-*oprF_*his following the manufacturer’s instructions.

### Recombinant OprF Purification

Overnight cultures (100 mL) of *E. coli* BL21 (DE3) transformed with pET28a-*oprf*_his vectors were inoculated into 2 L of lysogeny broth (LB) with 1% glucose and 50 μg/mL kanamycin. Cultures were grown to mid-log phase at 37 °C with shaking at 200 rpm and then induced with 1 mM IPTG for 20 h at 20 °C with shaking. Cells were pelleted at 2500 × *g* for 10 min at 4 °C and resuspended in 100 mL of Ni-NTA lysis buffer (50 mM NaH_2_PO_4_, 300 mM NaCl, 10 mM imidazole, pH 8; 50 mL/1 L culture) with 1 mg/mL lysozyme and 1× Complete Mini EDTA-free protease inhibitor (Roche). The suspension was incubated at 37 °C with shaking and then sonicated on ice (10 cycles of 30 s at 20% amplitude, with 30 s rest). Lysates were centrifuged (40 min at 16,000 × *g*, 4 °C) to separate the insoluble fraction, washed with 2% Triton X-100 in PBS, and then washed again with PBS alone. Recombinant OprF was extracted in 20 mL of 50 mM NaH_2_PO_4_, 300 mM NaCl, 10 mM imidazole, and 8 M urea (pH 8) and applied to a HisPur Ni-NTA Superflow Agarose resin (Thermo Fisher) in a gravity flow column. The resin was equilibrated with 10 mM imidazole, washed with 20 mM imidazole, and eluted with 350 mM imidazole. Urea and imidazole were removed by dialysis using a 10K MWCO SnakeSkin Dialysis Tubing (Thermo Fisher): 4 h in 2 M urea, 50 mM NaH_2_PO_4_, and 300 mM NaCl, followed by overnight dialysis in 50 mM NaH_2_PO_4_ and 300 mM NaCl with 2% glycine. The antigen was further purified by size exclusion chromatography on a HiLoad 16/60 Superdex 75 SEC column (AKTA Vivo). Pooled fractions were concentrated by centrifugation (4000 × *g*, 4 °C, 1 h) using an Amicon Ultra 15 mL centrifugal device with Ultracel 10 MWCO filter (Sigma-Aldrich). Protein concentration and endotoxin levels were measured using the Pierce BCA Protein Assay Kit and Pierce Chromogenic Endotoxin Quant Kit (Thermo Fisher), respectively.

### Immunization of Mice with rOprF and Adjuvants

Female C57BL/6J mice (6–8 weeks old) were purchased from Charles River (UK), randomly grouped (*n* = 7), housed in individually ventilated cages (3 or 4 per cage), and acclimatized for 2 weeks. Food and water were available *ad libitum*. Sigma Adjuvant System (SAS) was resuspended in buffer (50 mM NaH_2_PO_4_, 300 mM NaCl, pH 8) and mixed 1:1 with either the saline buffer alone or 50 μg/mouse of rOprF. SAS is a commercial adjuvant, and each dose contains 12.5 μg/mouse of MPLA from *Salmonella minnesota* and 12.5 μg/mouse of synthetic trehalose dicorynomycolate in 2% oil (squalene)-Tween 80-water. FP18 and FP20Rha were prepared in the same buffer as SAS and rOprF, with a final concentration of 0.59%/g (v/w) DMSO per dose. Mice were immunized subcutaneously and subsequently boosted twice, 2 weeks apart, with either 50 μg of rOprF alone; 50 μg of rOprF plus 25 μg of FP18; 50 μg of rOprF plus 25 μg of FP20Rha; or 50 μg of rOprF plus SAS (∼25 μg of active component) in 100 μL (Figure 2A).

### Serum IgG Antibodies Determination

Blood was collected from each mouse via cheek bleeds 1 week after the second booster for serological analysis and allowed to stand at 4 °C overnight before being centrifuged for 30 min to isolate sera, which were transferred to freshly labeled tubes and stored at −80 °C until required. Seroconversion was determined by ELISA as previously described.^53^ Briefly, microtiter plates were coated overnight with 100 μL of purified rOprF antigen (0.5 μg/mL) in 0.2 M sodium carbonate (pH 9.6) at 4 °C. Sera were added in five-fold dilutions to wells in triplicate and incubated for 2 h at room temperature. Plates were washed and then incubated with 100 μL of HRP-conjugated antimouse antibodies (IgG, IgG1, or IgG2c; 1:5000 in PBS with 1% FBS) for 60 min at room temperature. After washing, the TMB substrate (100 μL) was added, and the reaction was stopped with 2 M sulfuric acid before measuring absorbance at 450 nm. Sera from mice treated with FP18 or FP20Rha adjuvants (without an antigen) served as controls. Data were analyzed using GraphPad Prism 8.0.2 by calculating the area under the curve for each sample.

### Bacterial Challenge and Determination of Organ Colonization

Two weeks after the second booster, mice were challenged by oropharyngeal aspiration with 5.9 × 10^6^ CFU/mouse and culled after 24 h.^55^ Lungs, spleens, and stomach were aseptically harvested in PBS, weighed, and homogenized in a TissueLyser II (Qiagen) for 15 min at the maximum frequency (30 Hz/s) using 3.2 mm stainless steel beads. Organ homogenates were then serially diluted in PBS, plated onto LB agar in duplicate, and incubated at 37 °C, and the CFU was counted after 24 h.

### *Ex Vivo* Restimulation Assays and Flow Cytometry

Female C57BL/6J mice (6–8 weeks old; *n* = 7) were immunized subcutaneously, and after 14 days, the mice were humanely sacrificed. The individual spleens were aseptically harvested, mechanically disrupted, and filtered to obtain a single-cell suspension of splenocytes, as previously published.^53,54^ Then, an ammonium–chloride–potassium (ACK) lysis buffer was used to remove red blood cells. The splenocytes were counted using a Countess Automated Cell Counter (Invitrogen, Carlsbad, CA, USA). One million cells in triplicate (per mouse) were plated per well and stimulated for 60 h with 10 μg/mL of rOprF in a 96-well plate using 10% FBS RPMI medium and 1% P/S (penicillin–streptomycin). Between 5 and 6 h before harvesting the cells, 5 μg/mL of brefeldin A was added to block cell trafficking and increase the accumulation of intracellular cytokines. Cells were collected by centrifugation and stained for flow cytometry. Staining and flow cytometry of the cells were performed using published procedures,^53,54^ with a master mix of fluorophore-conjugated antibodies (50 μL per well; see Table S2). Flow cytometry was performed using a Beckman Coulter CytoFLEX LX (NUV full configuration). Quality control was conducted with Beckman Coulter Daily QC beads and IR Daily QC as per the manufacturers’ specifications. Data analysis was done with Beckman Coulter CytExpert v.2.4 software. Fluorescence minus one (FMO) control from splenocytes of a control mouse was used for gating strategies, and data were expressed as percentages of parent cells (Figures S5–S16).

### Statistical Analysis

Statistically significant differences between control and immunized mice were analyzed using the unpaired non-parametric Kruskal–Wallis test (*p*-value <0.05) when the data did not pass the Shapiro–Wilk normality test. Serology data were compared by one-way ANOVA. The Tukey multiple-comparison test following one-way ANOVA was performed to obtain adjusted *p*-values. For statistical comparisons of serology data, the areas under the ELISA titration curves (AUC) were compared by Brown–Forsythe and Welch one-way ANOVA tests with an α of 0.05. The Shapiro–Wilk normality test was also used to evaluate the data distribution of flow cytometry data. Statistically significant differences (*p*-value <0.05) were evaluated using a one-way ANOVA test for data with normal distribution or a Kruskal-Wallis test for data that did not follow a normal distribution.

## Supporting Information Available

(a) Additional methods; (b) antigen purification and characterization; (c) flow cytometry experimental set up and FMOs (PDF)

## Abbreviations

FMO,: fluorescence minus one
IFN,: interferon
IL,: interleukin
LPS,: lipopolysaccharide
MAPK,: mitogen activated protein kinase
MPLA,: monophosphoryl lipid A
NK,: natural killer
NKT,: natural killer T cells
NLRP3,: NOD-like receptor family pyrin domain containing 3
OprF,: outer membrane protein F
SAS,: Sigma Adjuvanted System
