# Supplementary material for: Monosaccharide-Based Synthetic TLR4 Agonist Enhances Vaccine Efficacy against Pseudomonas aeruginosa Challenge
Source: ACS Infect Dis. 2025 Mar 25;11(4):894–904. doi: 10.1021/acsinfecdis.4c00932 (PMC11998000; doi:10.1021/acsinfecdis.4c00932)
Supplement: Supplementary file 1 — id4c00932_si_001.pdf [file id4c00932_si_001.pdf]

Monosaccharide-based synthetic TLR4 agonist enhances vaccine efficacy against *Pseudomonas aeruginosa* challenge

Maite Sainz-Mejías[1], Chaoying Ma[1], Yueran Hou[1], Irene Jurado-Martin[1], Alessio Romerio,[2,] Ana Rita Franco[2], Mohammed Monsoor Shaik[2], Julen Tomás-Cortázar[1], Francesco Peri[2], Siobhán McClean[1]*.

[1] School of Biomolecular and Biomedical Sciences and Conway Institute of Biomolecular and Biomedical Research, University College Dublin, Belfield, Dublin 4, D04 V1W8, Ireland

[2] Department of Biotechnology and Biosciences, University of Milano-Bicocca, Piazza della Scienza, 2, 20126 Milano, Italy.

***Correspondence:**
Corresponding Author: Siobhán McClean
siobhan.mcclean@ucd.ie

Supplementary Information

15 pages; 3 tables; 17 figures.

Supplementary Method:

Purification of the recombinant OprF-His Protein:

The *oprf* gene was cloned into the pET28a expression vector with the His6 affinity tag at the 5' end of the polylinker to enable affinity purification (Figure S1). The gene fragment is flanked by BamHI and HindIII restriction enzyme sites, and is under the strong T7 promoter. The 6xHis tag is located in the N terminal domain (Figure S1).

Optimal recombinant OprF (rOprF) expression was observed 20 hours after induction with 1 mM IPTG at 20ºC (Figure S2). Following purification on a Ni-NTA column, the fractions containing eluted rOprF were pooled, concentrated and further purified by size exclusion chromatography (SEC), as indicated by a single large peak on chromatograms between fractions 25 and 30 representing the rOprF protein (Figure S3). and its purity was determined by SDS-PAGE (Figure S3). A pure fraction was achieved by observing the lack of other bands in the SDS PAGE gel (Figure S3C) or peaks during SEC. The rOprF eluted at a fraction volume of 40mL, suggesting a molecular weight of 80kDa, hence the dimerisation of the proteins. A smaller peak also eluted at a fraction volume of 60mL suggesting a molecular weight of 40kDa, thus the monomer of rOprF. The identification of the purified antigen was further confirmed by MS analysis (35 peptides matched; sequence coverage 78%) (Figure S3).

Supplemental tables:

Table S1. LPS quantification in rOprF sample

EU/mL	Absorbance		Average	Without blank			
	1	2					
**1**	1.055	1.028	1.042	0.698			
**0.5**	0.655	0.877	0.766	0.4225			
**0.25**	0.519	0.511	0.515	0.1715			
**0.1**	0.434	0.428	0.431	0.0875			
**0**	0.336	0.351	0.3435	0	**EU/mL**	**dil factor**	**EU/dose**
**OprF 1:5**	1.395	1.52	14.575	1.114	0.795	3,977	**0,20**

Table S2. Composition of the Ab master mix (BD Biosciences) used to label both intracellular and extracellular receptors and cytokines for their analysis by flow cytometry.

Target location	Ab target	Fluorophore	Reading channel	Dilution
Extracellular	CD3	BV786	V763	1:100
	CD4	BUV395	V405	1:200
	CD8	APC-H7	R763	1:100
	CD25	BV421	V450	1:100
	CD44	PE-Cy7	Y763	1:200
	CD49b	FITC	B525	1:100
	CD62L	BV480	V252	1:200
	γδ	R718	R712	1:100
Intracellular	IFN-γ	BV650	V660	1:100
	IL-4	BV605	V610	1:100
	IL-17A	PE-CF594	B610	1:100
	IL-22	PE	Y585	1:100
	TNF	BB700	B690	1:100
	FoxP3	AlexaFluor 647	R660	1:100

Table S3. Antigen-Specific Antibody Titers and IgG1/IgG2c Ratios.

Group	IgG Titers	IgG1 Titers	IgG2c Titers	IgG1/IgG2c
**OprF**	15625±0.11	15625±0.14	15625±0.17	1
**OprF+SAS**	78125±0.02	78125±0.03	78125±0.05	1
**OprF+FP18**	78125±0.02	78125±0.03	15625±0.17	5
**OprF+FP20Rha**	15625±0.11	78125±0.03	15625±0.17	5

<u>Supplementary Figures:</u>

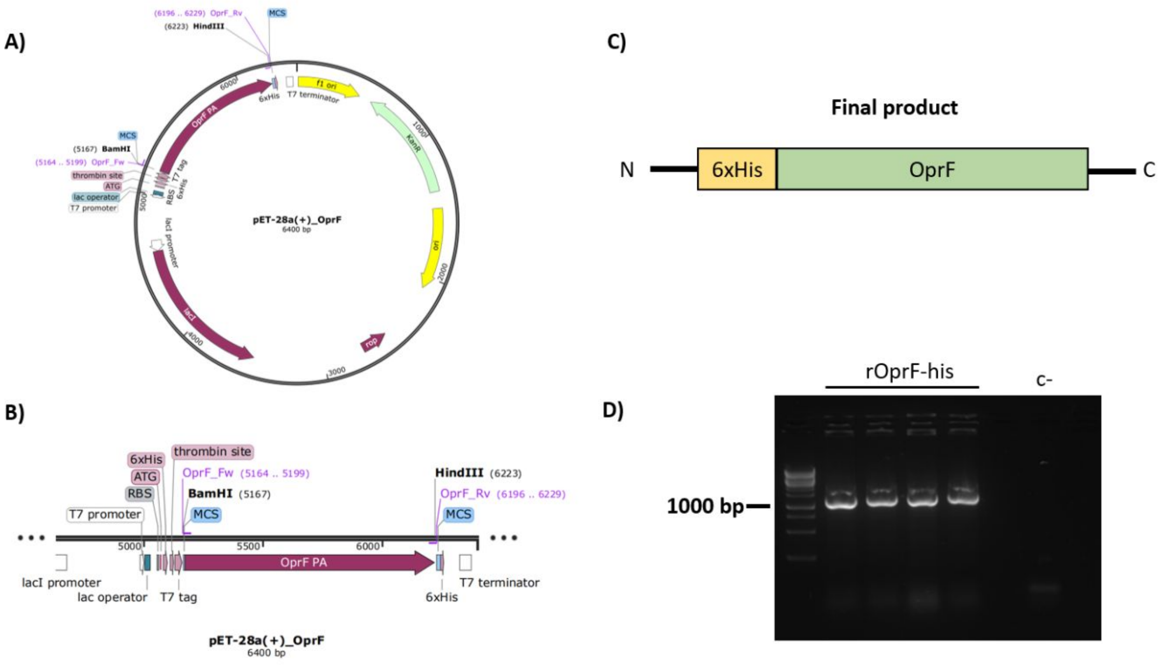

Figure S1. Expression of rOprF in a pET-28 a(+) vector. A) Map of the plasmid used for rOprF expression. B) Linear representation of the plasmid region where oprf was inserted (1050 bp). C) Representation of the final product obtained by inducing the expression of pET-28 a(+):: *oprf*_his. D) Colony-PCR of the *E. coli* BL21 (DE3) containing pET-28 a(+):: *oprf*_his indicating the successful insertion of the gene into the expression vector.

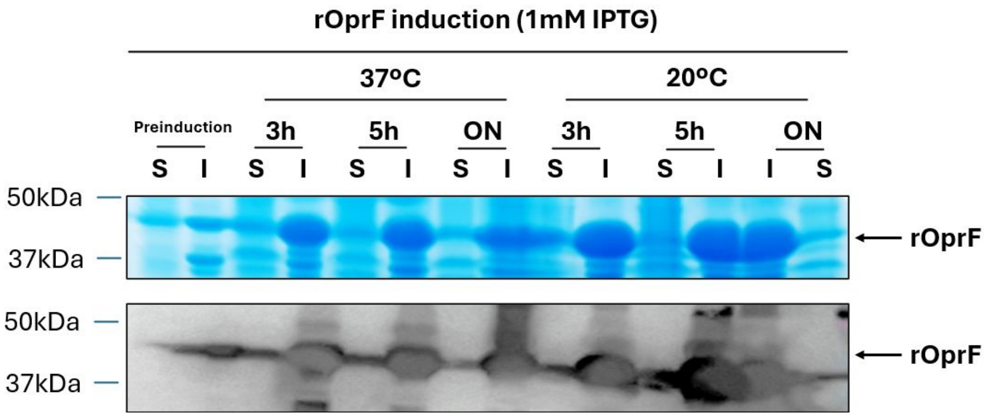

Figure S2. Optimisation of the rOprF expression in *E.coli* BL21 (DE3). Cultures of *E.coli* BL21 (DE3) pet28::*oprf* were induced with 1mM IPTG for 3h, 5h or overnight (ON) at either 37ºC or 20ºC. A sample before induction with IPTG was taken (pre-induction). The supernatants (S, Soluble fraction) were separated from the pellets (I, insoluble fraction). Western Blot analysis with an anti-6x histidine tag monoclonal antibody (Invitrogen, MA1-21315) confirmed the expression of rOprF.

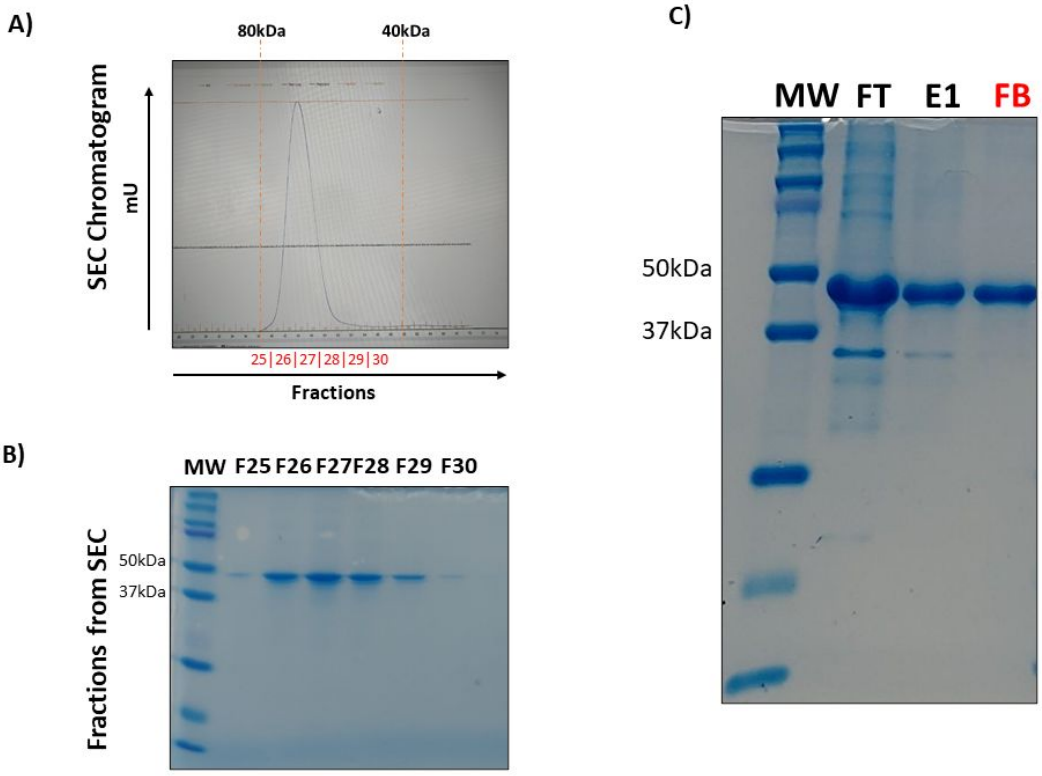

Figure S3. Purification of rOprF-his (41kDa) by nickel affinity column continued by size exclusion (SEC) chromatography. A) The purification of expressed and affinity-purified rOprF using a HiLoad® 16/600 Superdex® 75 pg column 16/60**.** Fractions collected representative of the large peak at a run volume of 40-52 mL (fractions 25-30). **B)** Purity of fractions 25-30 was visualised on 12% SDS gel. **C)** The purity and identity of the pooled SEC fractions were confirmed by SDS-PAGE. FT, Flow-through of the first purification by nickel affinity chromatography; E1, elution after nickel affinity chromatography; FB, final batch, after purification by SEC (pooled fractions 25-30).

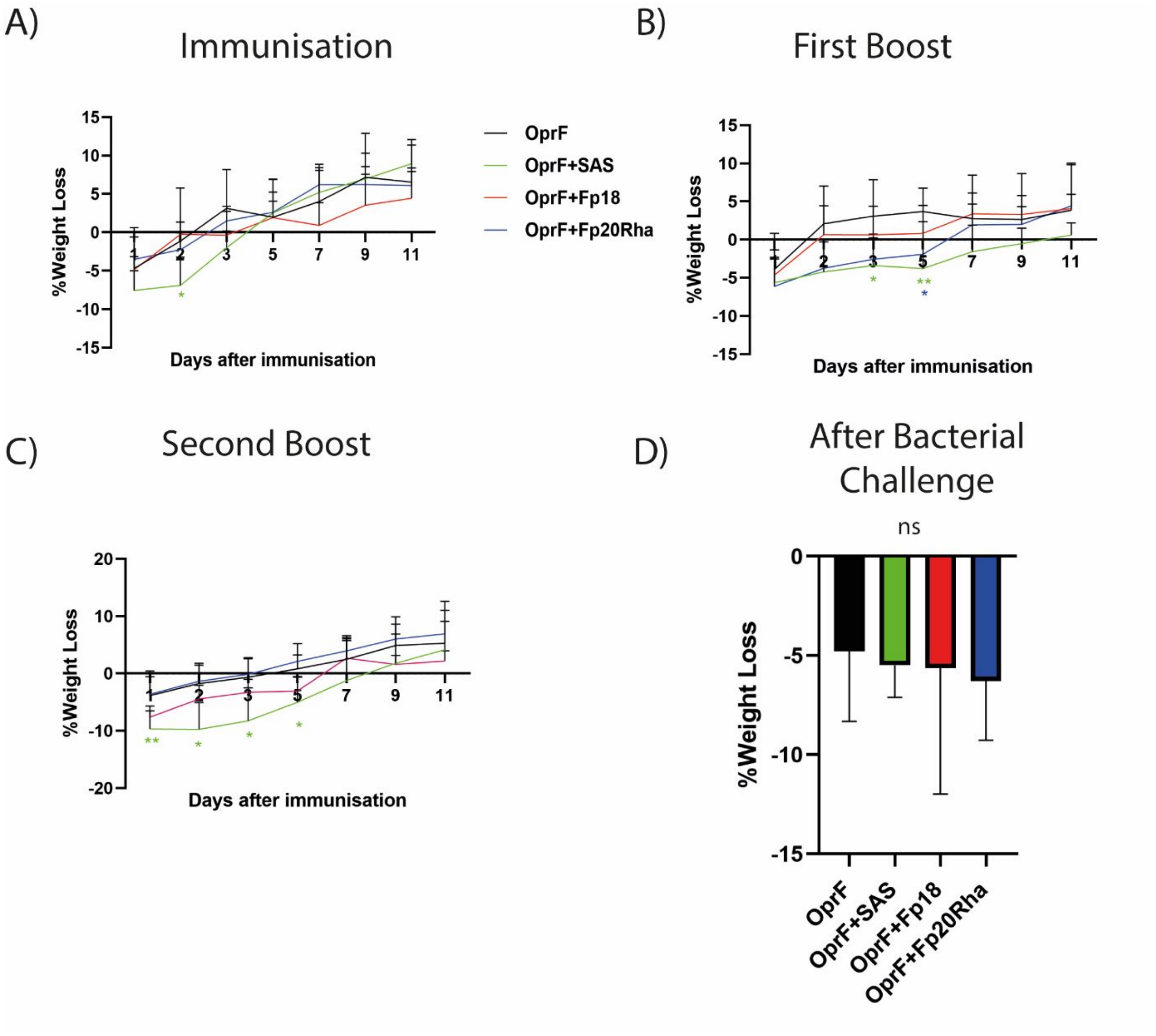

Figure S4. Weight loss during the mice immunisation study. A,B,C) The mean ± SD weight change in mice at each dose post-immunisation is shown (Immunisation, First Boost and Second Boost). D) Weight loss after bacterial challenge (mean ± SD).

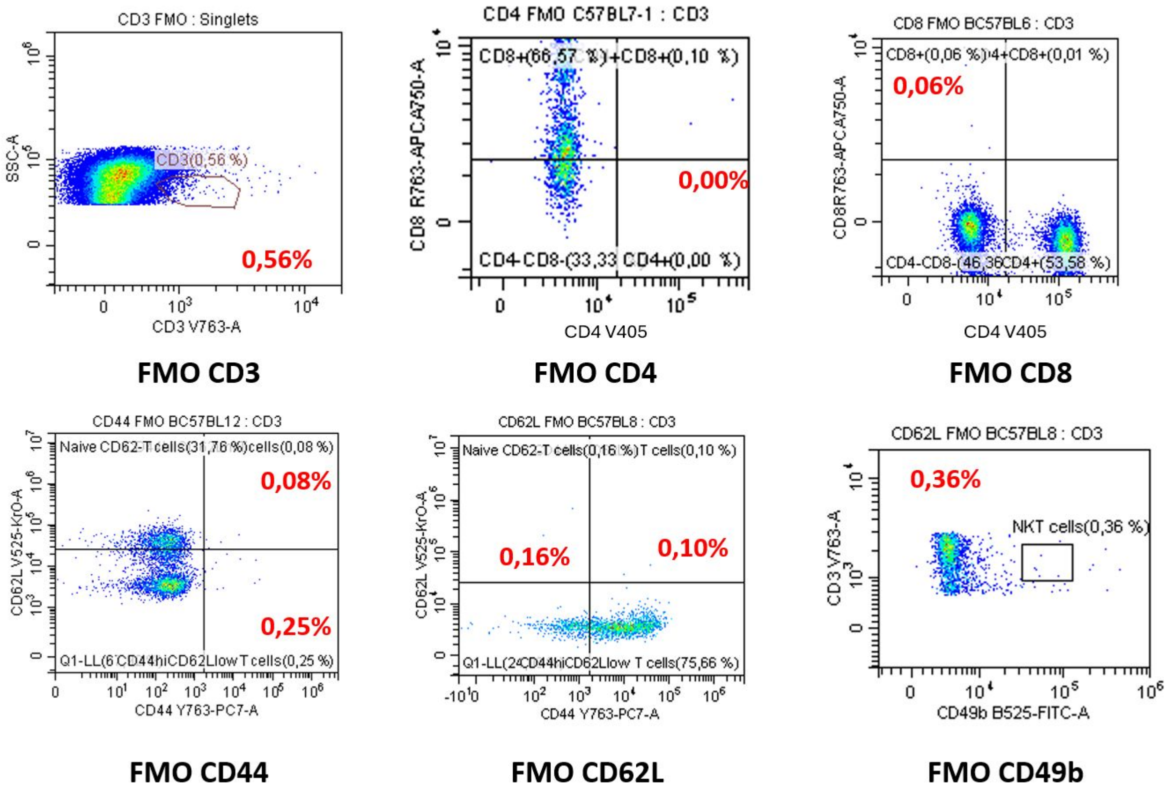

Figure S5. Fluorescence minus one (FMO) for each cell marker used in the flow cytometry analysis of the restimulated splenocytes after immunisation with rOprF adjuvanted with FP compounds. The percentage of the FMO is highlighted in red in each of the plots. The plots were achieved using the software CytExpert 2.5.

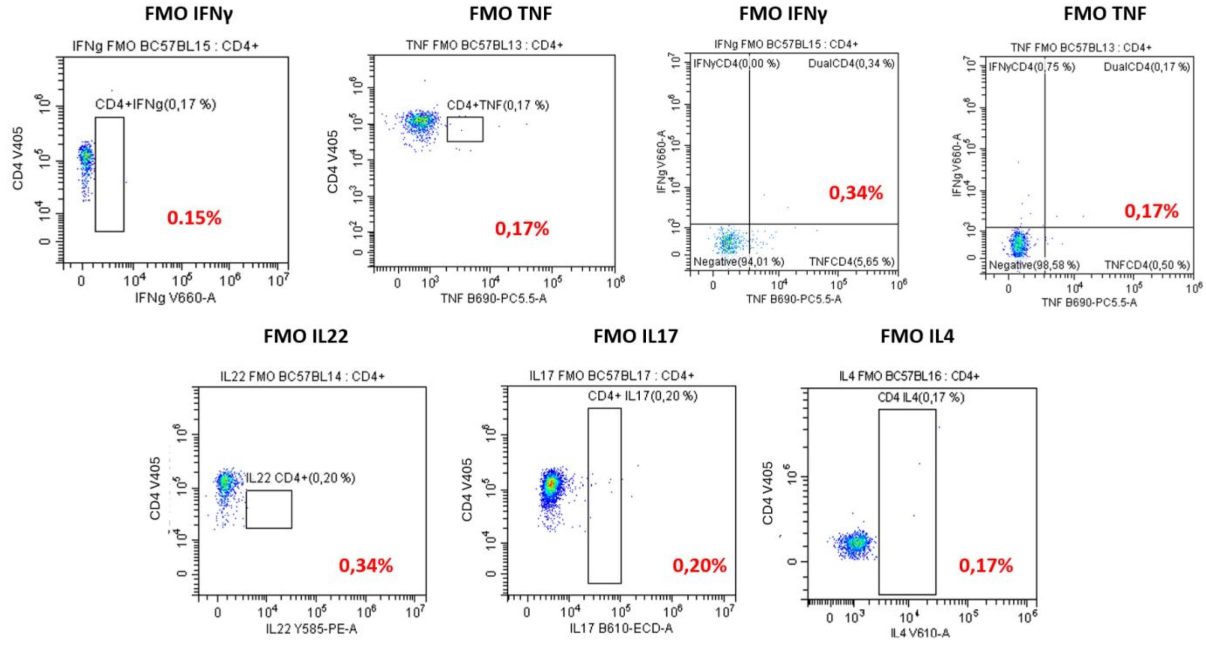

Figure S6. Fluorescence minus one (FMO) for each of the cytokines from $CD4^+$ T-cells in the flow cytometry of restimulated splenocytes after immunisation with rOprF adjuvanted with FP compounds. The percentage of the FMO is highlighted in red in each of the plots. The plots were achieved using the software CytExpert 2.5.

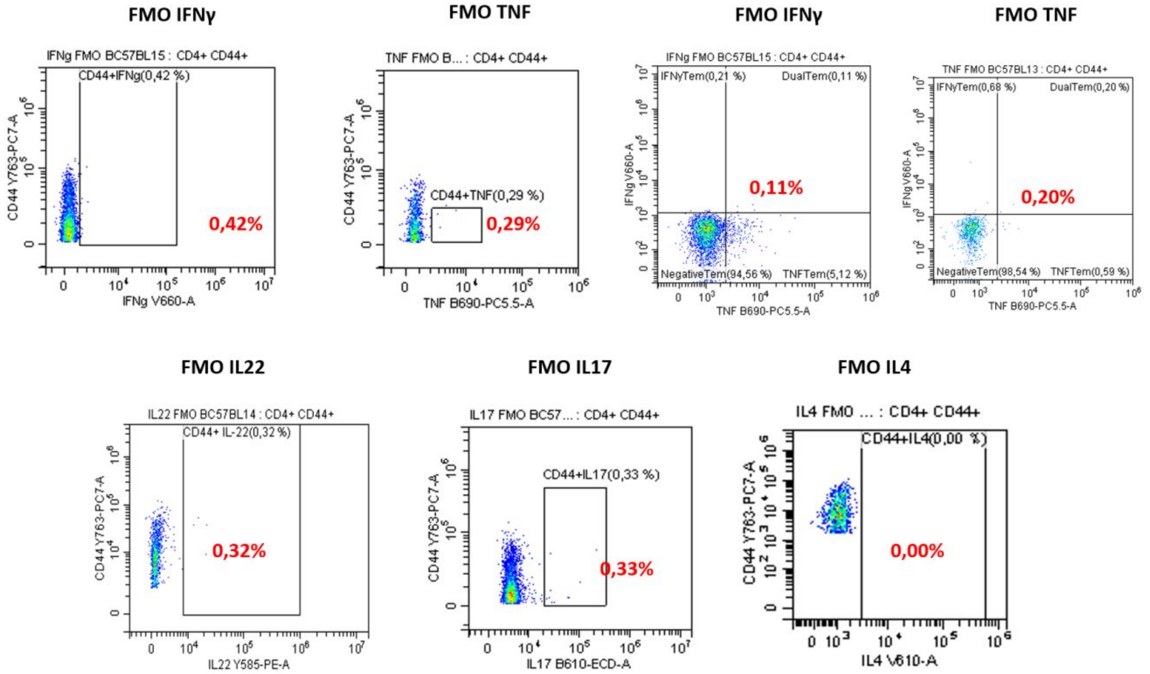

Figure S7. Fluorescence minus one (FMO) for each of the cytokines from $CD4^+CD44^{hi}$ T-cells in the flow cytometry of restimulated splenocytes after immunisation with rOprF adjuvanted with FP compounds. The percentage of the FMO is highlighted in red in each of the plots. The plots were achieved using the software CytExpert 2.5.

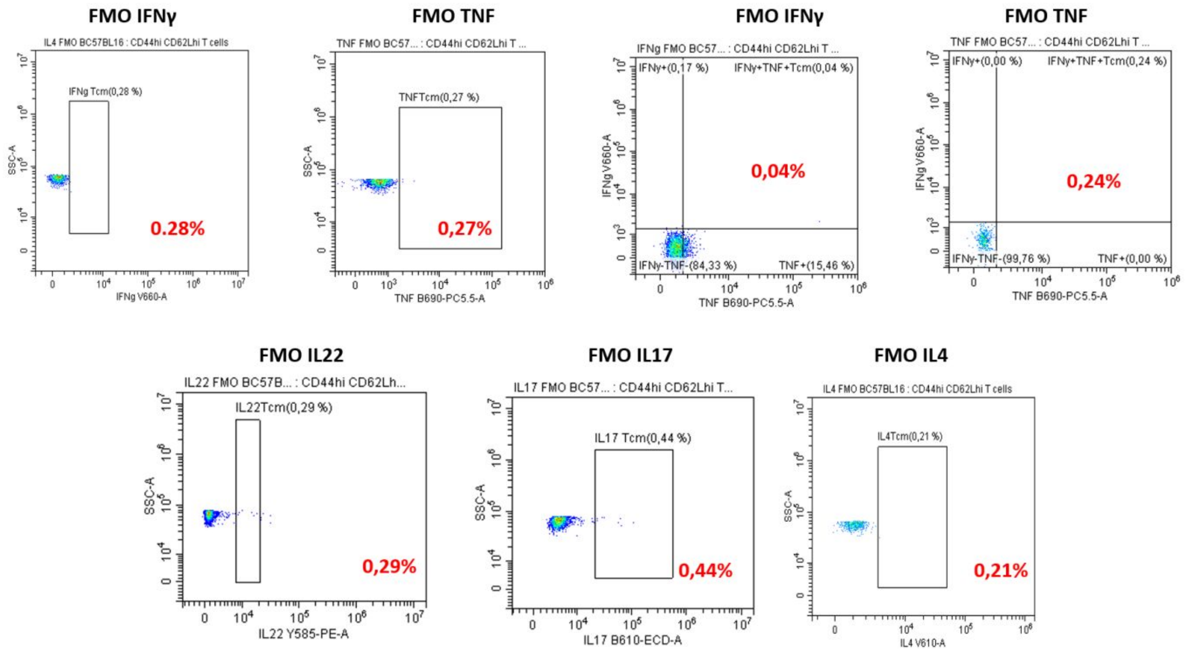

Figure S8. Fluorescence minus one (FMO) for each of the cytokines from CD4^{+}CD44^{hi} CD62L^{hi} T-cells in the flow cytometry of restimulated splenocytes after immunisation with rOprF adjuvanted with FP compounds. The percentage of the FMO is highlighted in red in each of the plots. The plots were achieved using the software CytExpert 2.5.

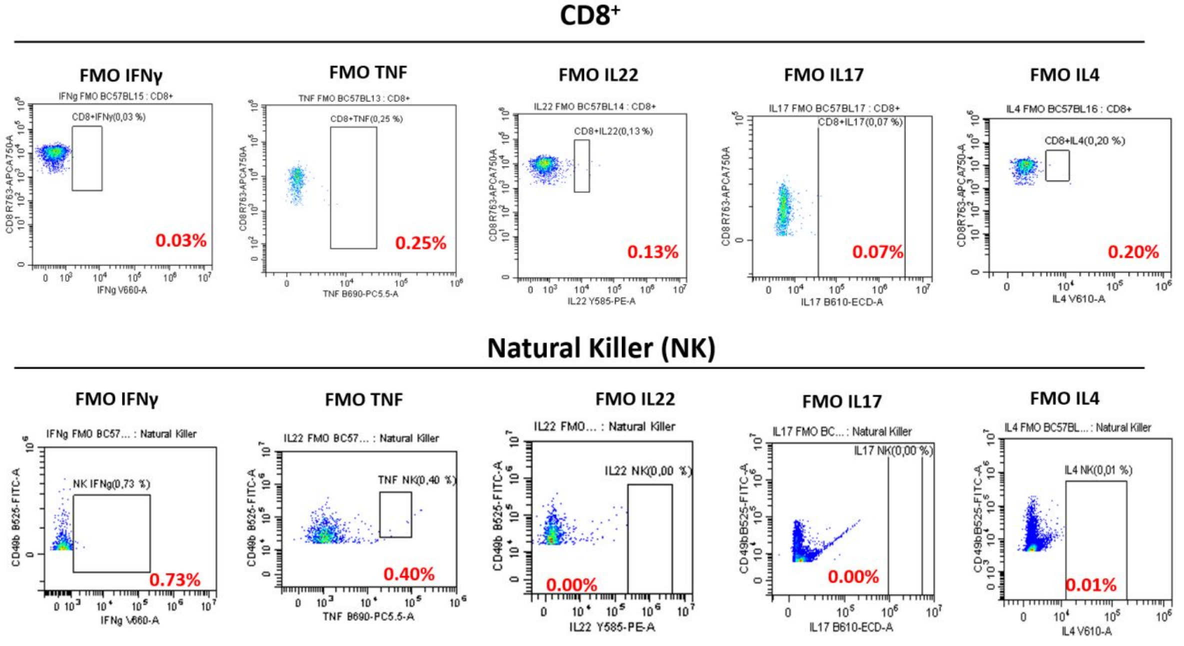

Figure S9. Fluorescence Minus One (FMO) for each of the cytokines from CD8^{+} T-cells and NK cells in the flow cytometry of restimulated splenocytes after immunisation with rOprF adjuvanted with FP compounds. The percentage of the FMO is highlighted in red in each of the plots. The plots were achieved using the software CytExpert 2.5.

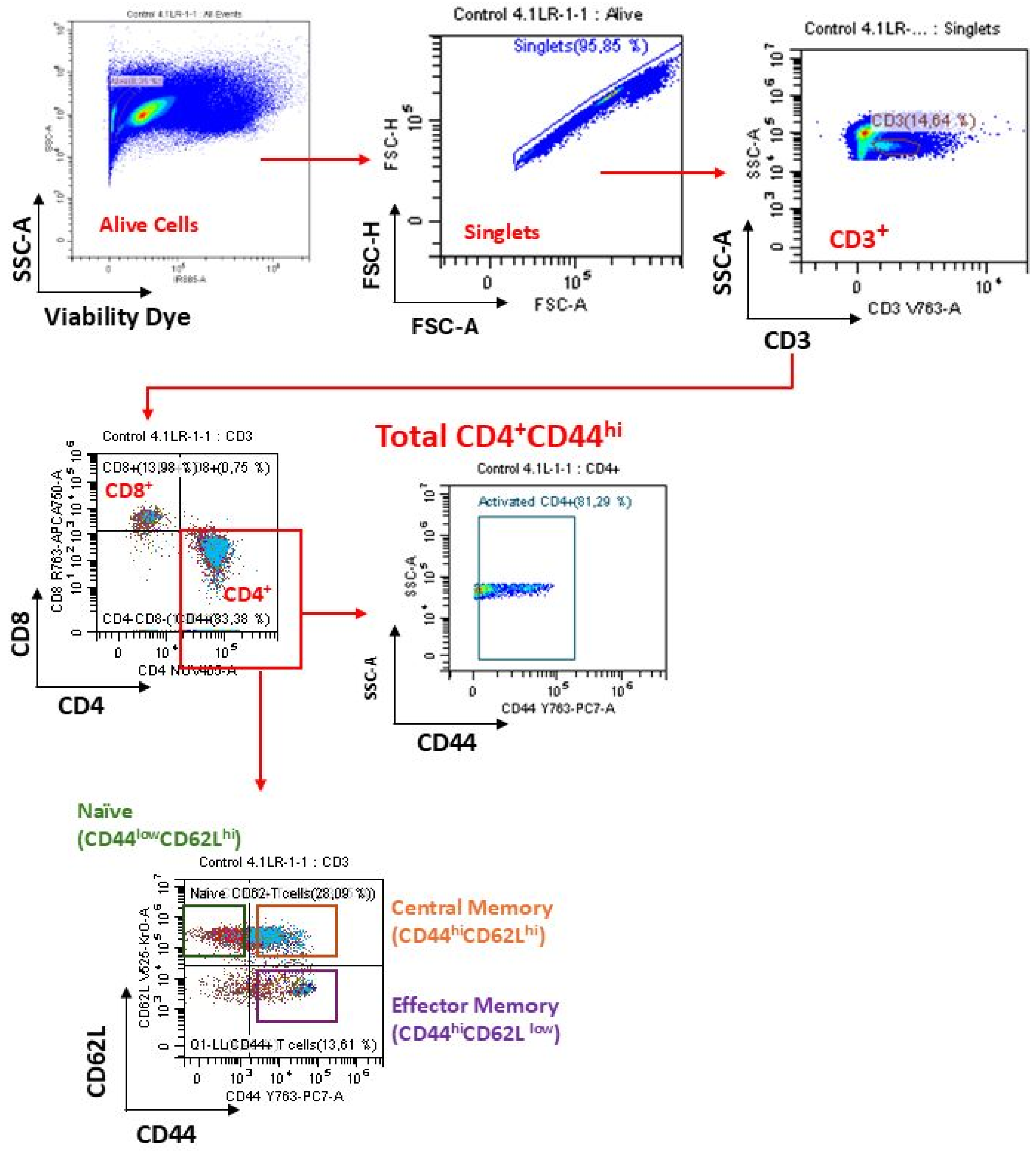

Figure S10. Gating strategy for CD3^{+}, CD8^{+} and CD4^{+} T-cell subpopulations. "Live cells" were identified by the viability dye, then singlets were selected and a CD3 marker was used to identify T-cells. The CD3^{+} T cells were then separated according to the CD8 and CD4 markers. And the CD4^{+} cells were further classified according to the CD44 and CD62L markers: Naïve (CD44^{low}CD62L^{hi}), effector memory (CD44^{hi}CD62L^{low}), central Memory (CD44^{hi}CD62L^{hi}), and activated (CD44^{hi}) . The black arrows highlight the axis of each plot and the red arrow the workflow used for the analysis

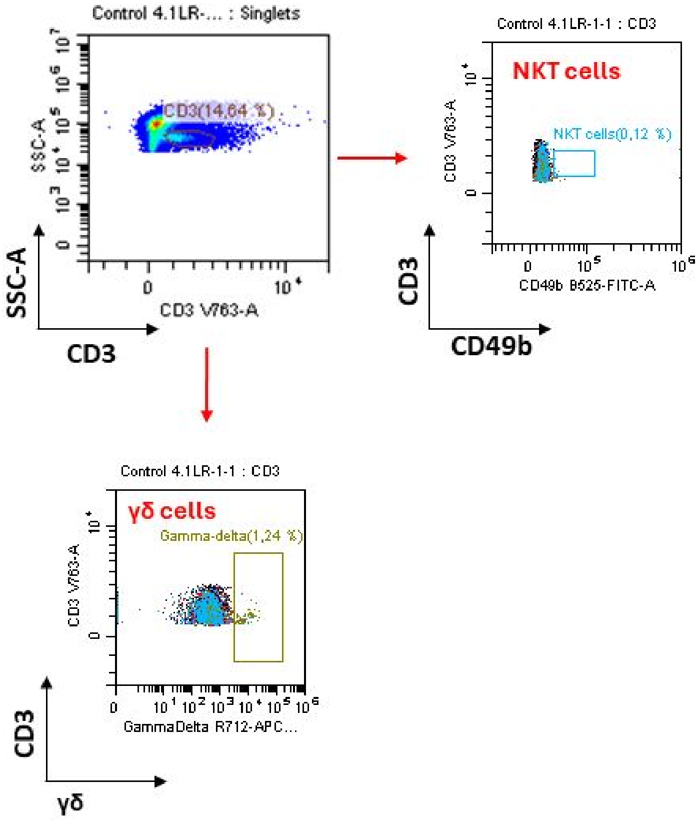

Figure S11. Gating strategy for γδ and NKT-cell subpopulations. The CD3⁺ T-cells were classified according to the CD49b (NKT cells) and γδ markers. The black arrows highlight the axis of each plot and the red arrow the workflow used for the analysis.

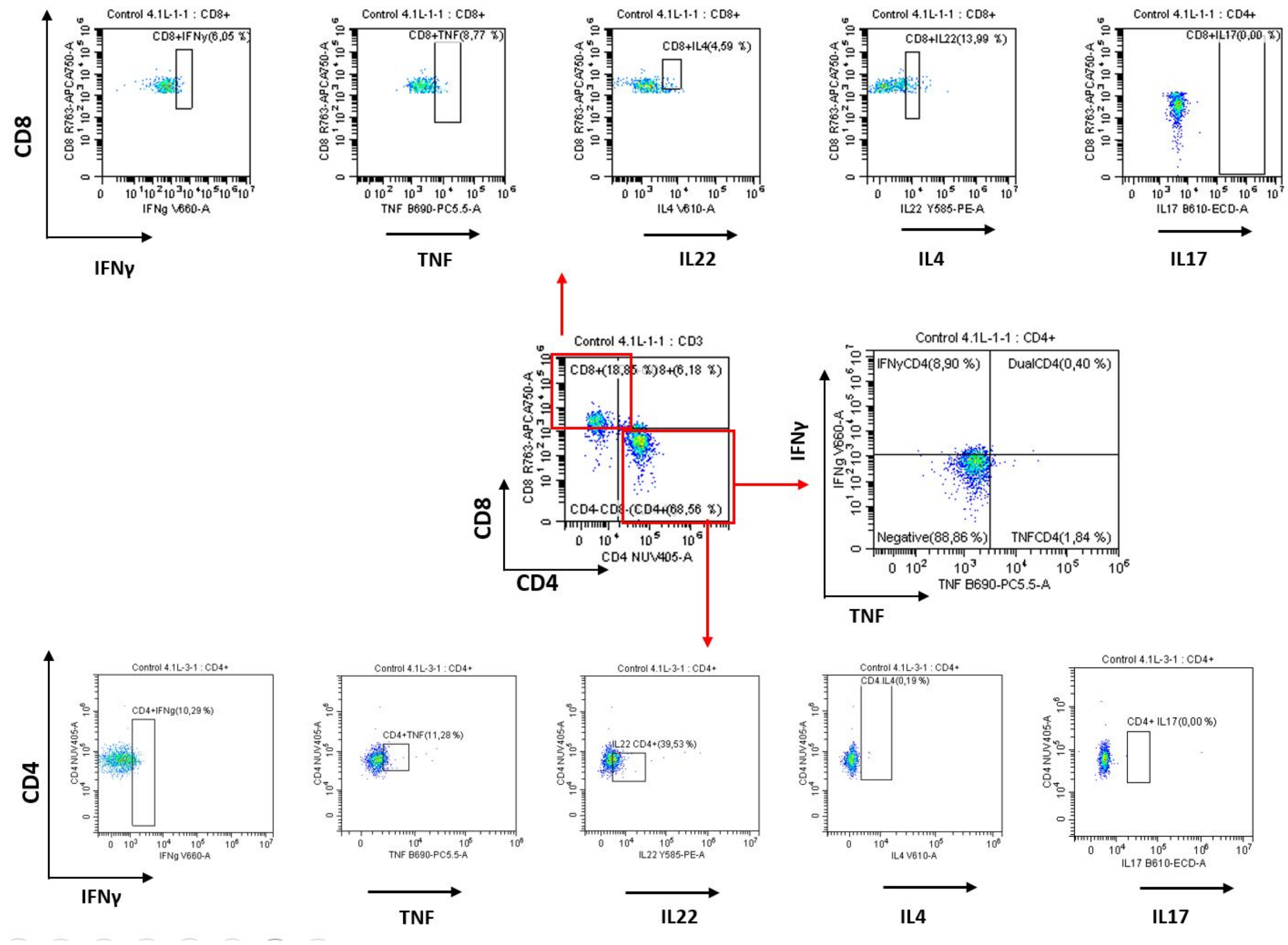

Figure S12. Gating Strategy for the cytokines expressed by CD8+ and CD4+ T-cell subpopulations. The CD8 and CD4 in combination with IFNγ, TNF, IL22, IL4 and IL17 markers were used to detect the stimulation of each of the cytokines from each of the T-cell subpopulation. The black arrows highlight the axis of each plot and the red arrow the workflow used for the analysis.

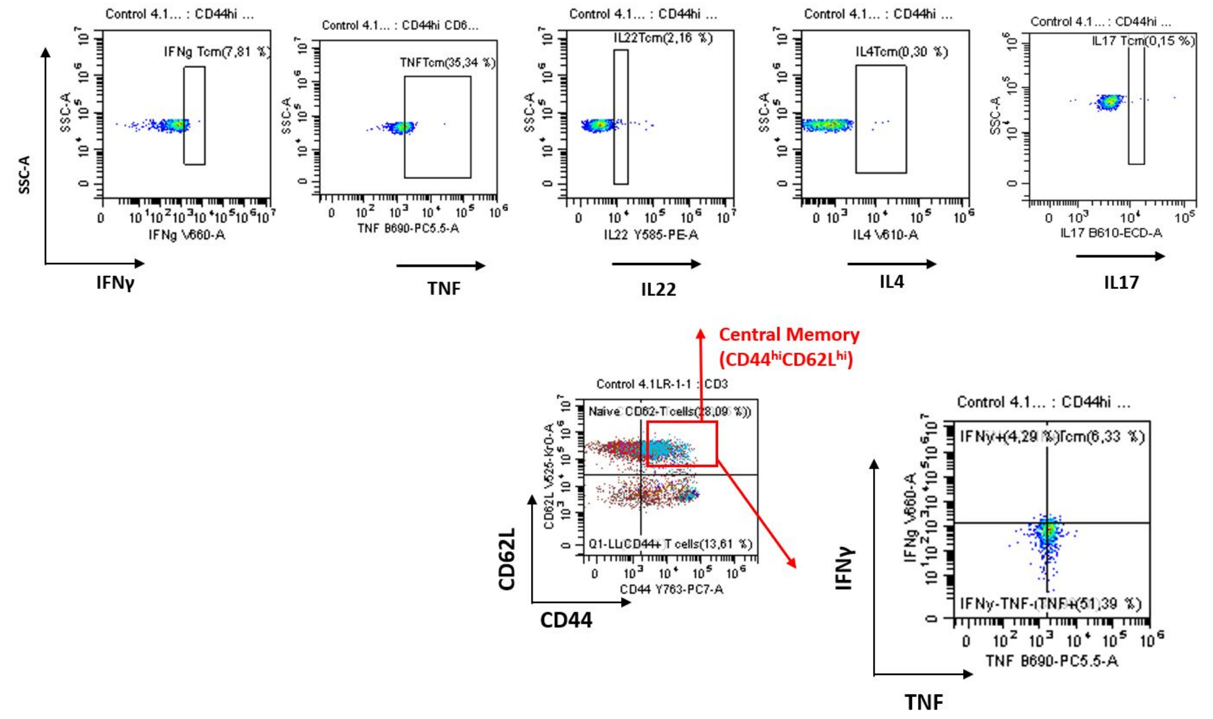

Figure S13. Gating Strategy for the cytokines expressed by central memory CD4⁺ T-cell subpopulations. The CD44 and CD62L in combination with IFNγ, TNF, IL22, IL4 and IL17 markers were used to detect the stimulation of each of the cytokines from the specific CD4⁺ T-cell subpopulation. The black arrows highlight the axis of each plot and the red arrow the workflow used for the analysis.

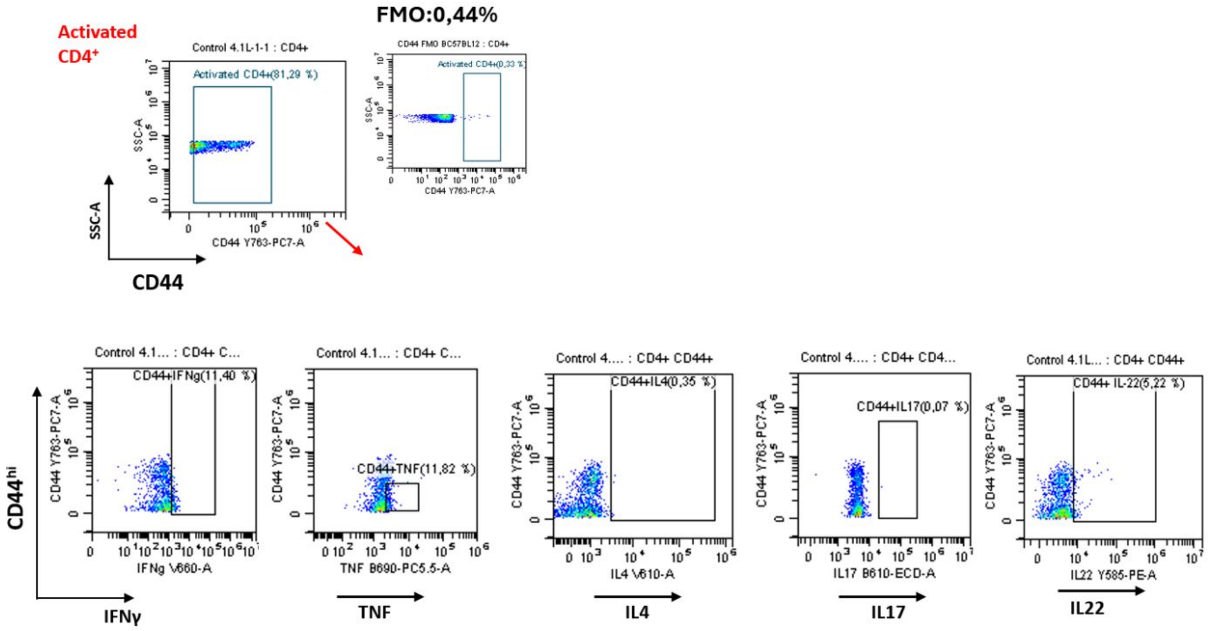

Figure S14. Gating Strategy for the cytokines expressed by CD4⁺CD44⁺ T-cell subpopulation. The CD44 in combination with IFNγ, TNF, IL22, IL4 and IL17 markers were used to detect the stimulation of each of the cytokines from the specific CD4⁺ T-cell subpopulation. The black arrows highlight the axis of each plot and the red arrow the workflow used for the analysis.

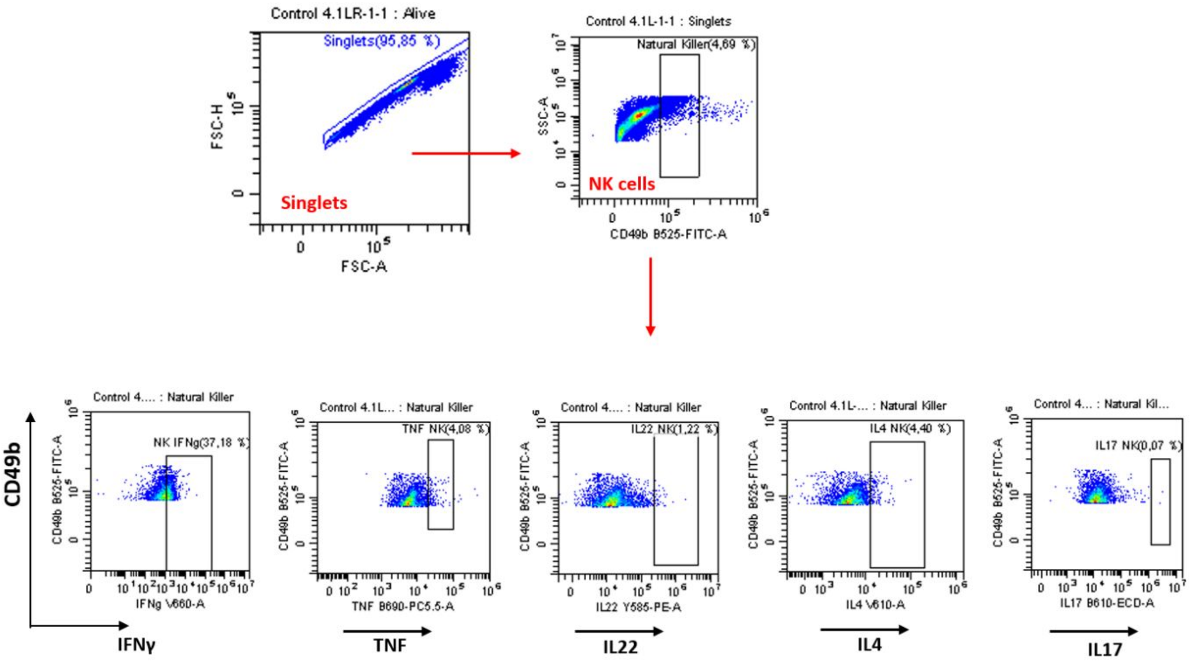

Figure S15. Gating Strategy for the cytokines expressed by NK cells. The CD49b marker was used to identify NK cells, and in combination with IFNγ, TNF, IL22, IL4 and IL17 markers to detect the stimulation of each of the cytokines by the NK cells. The black arrows highlight the axis of each plot and the red arrow the workflow used for the analysis.

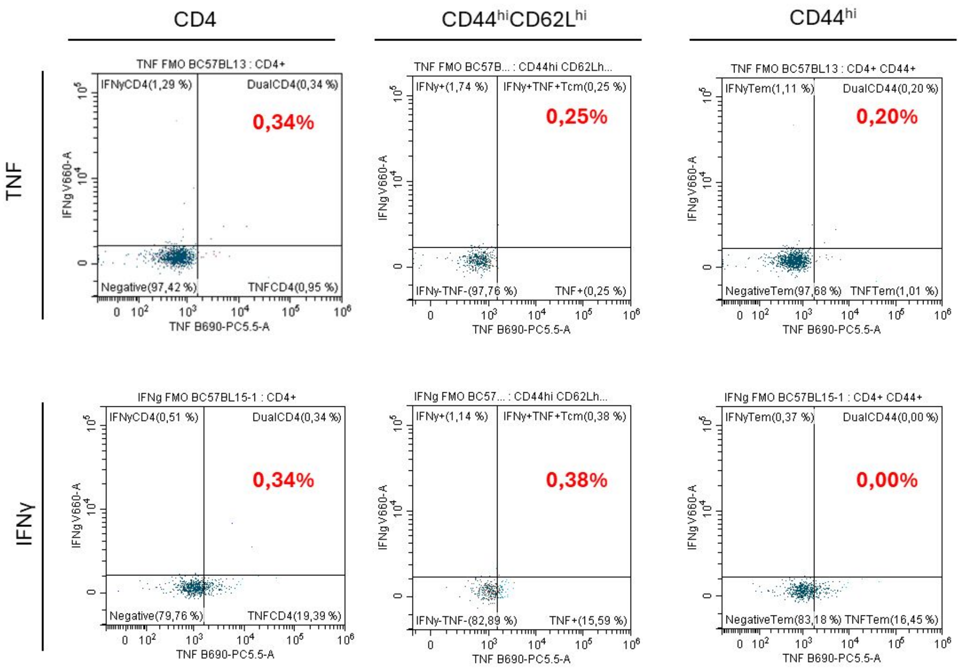

Figure S16. Fluorescence Minus One (FMO) for the evaluation of the dual expression of TNF and IFNγ from CD4$^+$, CD4$^+$CD44^{hi}CD62L^{hi} ,CD4$^+$CD44^{hi} T-cells. The percentage of the FMO is highlighted in red in each of the plots. The plots were achieved using the software CytExpert 2.5.

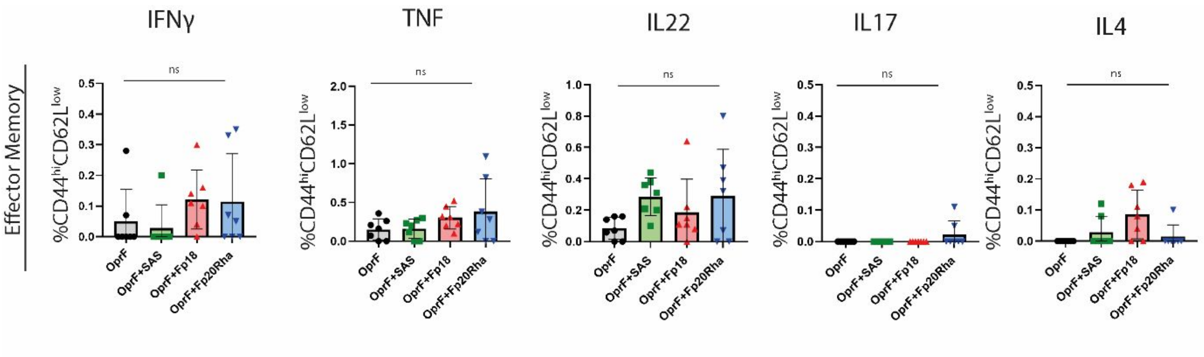

Figure S17. Evaluation of the cytokines elicited by CD4^{+}CD44^{hi}CD62L^{low} T-cell subtypes in restimulated splenocytes following immunisation with OprF adjuvanted with FP18 or FP20Rha. Percentages of effector memory CD4^{+} T cells expressing IFN-γ, TNF, IL22, IL17 or IL4 cytokines. Each dot in the graphs indicates the mean of technical triplicates of splenocytes from individual mice (n=7) as follows: splenocytes from mice immunised with rOprF (black circles); rOprF OprF+SAS (green squares); rOprF+FP18 (red triangles); or rOprF+ FP20Rha (blue triangles).
